# Preferred Reporting Items for Resistance Exercise Studies (PRIRES): A Checklist Developed Using an Umbrella Review of Systematic Reviews

**DOI:** 10.1186/s40798-023-00640-1

**Published:** 2023-12-01

**Authors:** Ting-Yu Lin, Ting-Yu Chueh, Tsung-Min Hung

**Affiliations:** Department of Physical Education and Sport Sciences, National Taiwan Normal University, No. 162, Section 1, Heping East Road, Da’an District, Taipei City, 106 Taiwan

**Keywords:** Resistance training, Sports, Kinesiology, Athletes, Research method

## Abstract

**Background:**

The issues of replication and scientific transparency have been raised in exercise and sports science research. A potential means to address the replication crisis and enhance research reliability is to improve reporting quality and transparency. This study aims to formulate a reporting checklist as a supplement to the existing reporting guidelines, specifically for resistance exercise studies.

**Methods:**

PubMed (which covers Medline) and Scopus (which covers Medline, EMBASE, Ei Compendex, World Textile Index, Fluidex, Geobase, Biobase, and most journals in Web of Science) were searched for systematic reviews that comprised the primary studies directly comparing different resistance training methods. Basic data on the selected reviews, including on authors, publication years, and objectives, were summarized. The reporting items for the checklist were identified based on the objective of the reviews. Additional items from an existing checklist, namely the Consensus on Exercise Reporting Template, a National Strength and Conditioning Association handbook, and an article from the EQUATOR library were incorporated into the final reporting checklist.

**Results:**

Our database search retrieved 3595 relevant records. After automatic duplicate removal, the titles and abstracts of the remaining 2254 records were screened. The full texts of 137 records were then reviewed, and 88 systematic reviews that met the criteria were included in the umbrella review.

**Conclusion:**

Developed primarily by an umbrella review method, this checklist covers the research questions which have been systematically studied and is expected to improve the reporting completeness of future resistance exercise studies. The PRIRES checklist comprises 26 reporting items (39 subitems) that cover four major topics in resistance exercise intervention: 1) exercise selection, performance, and training parameters, 2) training program and progression, 3) exercise setting, and 4) planned vs actual training. The PRIRES checklist was designed specifically for reporting resistance exercise intervention. It is expected to be used with other reporting guidelines such as Consolidated Standards of Reporting Trials and Standard Protocol Items: Recommendations for Interventional Trials. This article presents only the development process and resulting items of the checklist. An accompanying article detailing the rationale for, the importance of, and examples of each item is being prepared.

**Registration:**

This study is registered with the EQUATOR Network under the title “Preferred Reporting Items for Resistance Exercise Studies (PRIRES).” PROSPERO registration number: CRD42021235259.

**Supplementary Information:**

The online version contains supplementary material available at 10.1186/s40798-023-00640-1.

## Key Points

- The PRIRES checklist comprises 26 reporting items (39 subitems) that cover four major topics in resistance exercise intervention: 1) exercise selection, performance, and training parameters, 2) training program and progression, 3) exercise setting, and 4) planned vs actual training.
- The reporting items for the checklist were identified based on the objective of 88 included systematic reviews. Additional items from the Consensus on Exercise Reporting Template (CERT), a National Strength and Conditioning Association (NSCA) handbook, and an article from the EQUATOR library, were incorporated into the final reporting checklist.
- PRIRES checklist is expected to be used with other reporting guidelines such as Consolidated Standards of Reporting Trials (CONSORT) and Standard Protocol Items: Recommendations for Interventional Trials (SPIRIT).

**Supplementary Information:**

The online version contains supplementary material available at 10.1186/s40798-023-00640-1.

## Introduction

Resistance training is a potent as well as cost-effective non-pharmaceutical intervention for improving muscular and physical function. Research has shown that muscular strength can be improved by up to 50% in healthy older adults after one year of resistance training [1(R3)]. A recent analysis suggested that resistance training provides the best value for money for fall prevention compared to other unimodal and multimodal interventions [2(C)]. However, though generally beneficial, the effectiveness of resistance training is largely determined by parameters such as training intensity and frequency.

Selecting training parameters according to the training purpose is necessary for achieving optimal results. Although organizations such as the National Strength and Conditioning Association (NSCA) have developed some principles for resistance training, e.g., performing ≤ 6 repetitions for maximizing muscle strength gain [3(T15.11)], the influences of many other training parameters, such as optimal time under tension for increasing muscle hypertrophy, require further investigation. However, without complete reporting of the intervention, it is difficult to compare different primary studies and draw conclusions about preferred resistance training methods.

The issues of replication and scientific transparency have been raised in many research fields including psychology [4(T1)], social science [5(Pg638)], and medicine [6–8]. Potential means to address the replication crisis and enhance research reliability include trial registration [9(Sec3.2)], publishing the protocol before data collection, the registered reports [9 (Sec. 3.3), 10], a results-free peer review [11, 8, 12], conducting replication studies [4–6], and improving transparency [7, 13].

Although the Consolidated Standards of Reporting Trials (CONSORT) [13, 14] and Consensus on Exercise Reporting Template (CERT) [15] have been published to enhance the reporting quality of randomized controlled trials (RCTs) and exercise interventional studies, respectively, a supplementary preferred reporting checklist can further improve the reporting quality of resistance exercise studies. For instance, there were concerns regarding the reported resistance exercise method and program in 7 of the 11 studies summarized in our previous study [16(I3, T1)]. Specifically, of these 7 studies, three failed to report basic items related to resistance exercise, i.e., repetition, intensity, and rest intervals. In addition, some items regarding the resistance exercise such as the rest interval between sets and exercises and the order of exercises, which had been reported in our previous study [16, 13, 14] and CERT [15] checklists. Thus, a supplementary reporting checklist for resistance exercise studies can be beneficial to future research.

To overcome the limitations of the Delphi technique, which has been used to develop existing reporting checklists such as CONSORT [17(M)], an umbrella review was applied in this study. The Delphi technique was first proposed by Norman Dalkey and Olaf Helmer in the 1950s to develop consensus among experts [18(I3)]. Although it can provide some information, it suffers from several methodological disadvantages that can be avoided using the newer umbrella review technique. An umbrella review is a tertiary research design (in contrast to primary research such as RCTs and secondary research such as systematic reviews) [19(Pg5-6)] that emerged at the beginning of the twenty-first century. This design enables systematic data collection and synthesis on a broad issue, which is impractical for a traditional systematic review. The following are several major comparisons between these two research methods that are of consequence to our study. First, expert opinions in the Delphi technique are ranked the lowest in the evidence hierarchy, as opposed to the umbrella review which is considered the highest [19(F2.1)]. Second, concerns have been raised that the Delphi technique is not fully “systematic.” For instance, Humphrey-Murto and de Wit criticized the Delphi method for the ambiguity of its methodology, poor reporting quality, and the presence of little to no empirical evidence to support best practices in the consensus development stages [20(Pg136)]. Third, regarding the advantages of the proposed checklist, as a supplement to CONSORT, it will need to be updated regularly and in timely fasion for it to function optimally. The feasibility of rapidly developing and updating an umbrella review will be an advantage over the time-consuming Delphi technique. A detailed discussion of the pros and cons of the Delphi technique is beyond the scope of this protocol. An integrative introduction to the umbrella review has been edited by Biondi-Zoccai [19]. The limitations of the Delphi technique in methodology, process, results, and conclusion have been reviewed by Vernon [21], and the disadvantages of this technique, including researcher bias and shortcomings, unethical behavior caused by anonymity, and debates over the method rather than the topic, have been discussed by Avella [22].

After the search, we recognized that an article by Coratella [23], also published in *Sports Medicine Open,* shared a similar purpose to the PRIRES project. However, there were some differences between these two works. Coratella provided a checklist focusing on within-exercise variables [23], instead of a checklist for the entire resistance training program, and provided his valuable insight in a narrative review format. In contrast, we aimed to develop a checklist for the entire resistance training program with an umbrella review method and therefore preregistered this project with both EQUATOR in 2020 and PROSPERO in 2021. We believe that the PRIRES can broade in 2021. We believe that the PRIRES can broaden the scope of Coratella’s checklist. Even if some reporting suggestions might overlap conceptually between PRIRES and the article by Coratella, the methodological processing in generating these recommendations differed substantially.

There are two major differences in research methods between PRIRES and the excellent work by Coratella [23]. First, the research purposes are not the same. Coratella provided his insight and reporting recommendations (8 reporting items) focusing on the “within-exercise” variable, while PRIRES is a project for developing a complete checklist for resistance training interventions including between-exercise variables, training progress, and unconventional training methods (e.g., blood flow restriction).

The﻿ second major difference is the methodological approach to constructing the checklist. Although the PRIRES is not free from the authors’ subjective perspective (e.g., item 4c “relative intensity” was retrieved from the NSCA handbook based on our knowledge), a significant amount of effort was made to ensure this checklist was as objective as possible by developing the item extraction protocol, and as inclusive as possible by conducting a systematic search in two databases. The umbrella review and the narrative review are both important research formats and the reporting recommendations derived from these two different methods, even if they are similar conceptually, have their own merits.

This study aims to construct a reporting checklist as a supplement to the existing reporting guidelines such as CONSORT [13, 14], specifically for resistance exercise studies. A preferred reporting items checklist developed using umbrella review methods promises to be more systematic and provides a higher level of evidence than those developed using the Delphi technique. In order to show how the research question developed, the original objective and rationale, which were written before the results were known, are provided in Additional file 1: Original Rationale and Objective.

## Methods

This study was reported according to the reporting checklist for umbrella reviews published by Onishi and Furukawa in 2016 [24(T13.1)]; see Additional file 2: Umbrella Review Reporting Checklist.

### Inclusion Criteria

1. Systematic reviews that comprised primary studies directly comparing different resistance training methods.
2. Resistance exercise was the primary intervention.
3. There were no limitations on the characteristics of the participants recruited.

### Exclusion Criteria

1. Systematic reviews that involved other types of exercise, such as concurrent training (aerobic + resistance exercises), or other interventions (e.g., diet and supplements).
2. Reviews not published in full and/or not published in English.
3. Reviews that included animal studies.
4. Reviews that included studies of resistance training targeting respiratory or oral muscles.

### Search Strategies and Information Sources

Systematic reviews that investigate the effects of different resistance exercise parameters, such as the types of resistance exercise and training frequency, were identified by searching PubMed (covers Medline) and Scopus (covers Medline, EMBASE, Compendex, World Textile Index, Fluidex, Geobase, and Biobase [25(T1)] and most journals in Web of Science [26(F2)]) using the following keywords:

“resistance exercise*” or “resistance train*” or “weight exercise*” or “weight train*” or “weight bear*” or “weight-bear*” “weightlift” or “weight lift*” or “strength train*” or “strength exercise*” or “power train*” or “power exercise” or “explosive exercise*” (in Title/Abstract).

AND

Systematic or meta (in Title).

The search syntax is outlined in Additional file 3: Syntax of Literature Search.

### Data Selection and Collection Process

The titles and abstracts retrieved from different databases were loaded into Endnote 20 to remove duplicates automatically. The authors TYL and TYC independently screened all titles and abstracts against the selection criteria. The full-text screening was performed if the titles and abstracts indicated that the studies met the criteria or if there was any uncertainty. Multiple reports were checked by searching the first author’s name in Endnote 20 (Clarivate Analytics, Philadelphia, PA, USA) and the registration ID. If multiple reports were found, the most detailed one was included. If disagreements could not be resolved via discussion, the author TMH was consulted.

### Reporting Items

Basic data on the included systematic reviews, including authors, publication years, and objectives, were extracted and tabulated. The reporting items were identified based on the objectives of the reviews. Additional information was sought from relevant published guidelines such as the CERT [15] from the EQUATOR library to build this reporting checklist for resistance exercise studies. The quality of the systematic reviews was not judged because this was beyond the scope of this umbrella review.

## Results

**Fig. 1:**
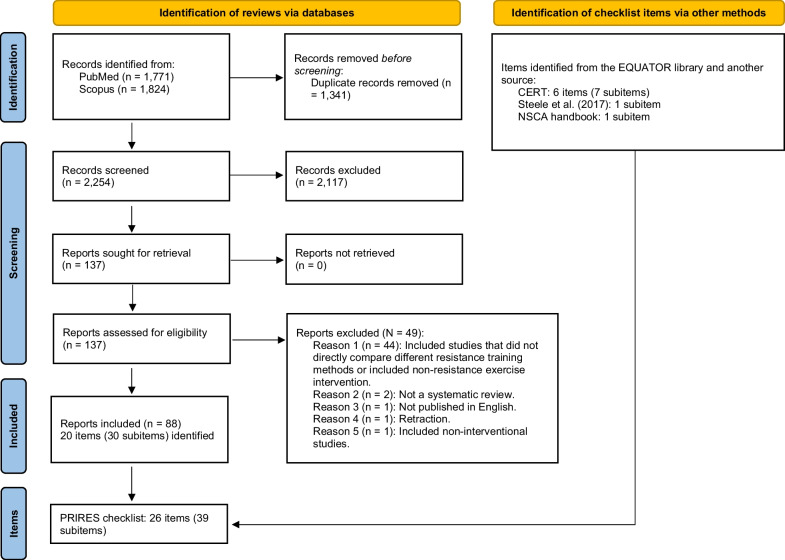
Modified PRISMA 2020 flowchart. CERT: Consensus on Exercise Reporting Template; NSCA: National Strength and Conditioning Association; PRIRES: Preferred Reporting Items for Resistance Exercise Studies; PRISMA: Preferred Reporting Items for Systematic Reviews and Meta-Analyses

Our database search retrieved 3,595 relevant records (May 5, 2022). After automatic duplicate removal by EndNote 20, the titles and abstracts of the remaining 2,254 records were manually screened by the authors TYL and TYC independently. One hundred and thirty-seven articles were successfully retrieved and their full texts were screened by TYL. Finally, 88 studies were included in the review; see Fig. 1 for the modified PRISMA 2020 flow diagram.

The 49 records excluded after the full-text review and the reasons for their exclusion are listed in Additional file 4: Records Excluded After the Full-Text Review and summarized in Fig. 1. The most common reason for exclusion was that the reviews included studies that did not directly compare different resistance training methods or included non-resistance exercise interventions (*n* = 44).

Table 1 shows all 26 reporting items (39 subitems) extracted. Twenty items (30 subitems) were identified from the included systematic reviews based on their research objectives (see Additional file 5: Included Reviews and Research Questions); six items (seven subitems) were adapted from the CERT reporting checklist; one subitem was retrieved from an article in the EQUATOR library, which aimed to clearly define a set endpoint in resistance exercise [27(T3)], and one subitem was identified from a National Strength and Conditioning Association (NSCA) handbook [3(T15.17)].

**Table 1:** Preferred Reporting Items for Resistance Exercise Studies (PRIRES) checklist

Topic	Item #	Checklist item	References
**Exercise selection, performance, and training parameters (a flowchart is recommended)**			
Exercise selection	1a	Provide the name and movement description of every exercise and report the exercises performed in each training session	Kassiano et al. [34]
	1b	Provide enough information for readers to know whether the exercises were free-weight or machine-based, and unilateral or bilateral	Free-weight vs machine-based Heidel et al. [35] Unilateral vs bilateral Moran et al. [36]
	1c*	Report the range of motion of each exercise	Schoenfeld and Grgic [37] Pallarés et al. [38]
Training frequency and length	2	Report the training frequency and length (e.g., 3 sessions/week × 12 weeks)	Schoenfeld et al. 2016 [39] Ralston et al. [40] Schoenfeld et al. [41] Grgic et al. [42] Ralston et al. [43] Schoenfeld et al. [44] Androulakis-Korakakis et al. [45] Baz-Valle et al. [46] Cuthbert et al. [47] Baz-Valle et al. [48]
Warm-up and movement descriptions	3	Report the content of the warm-up and transition (minutes) before the main resistance exercise training	Ribeiro et al. [49]
Intensity	4a*	Report the absolute training intensity in % 1RM (1 repetition max), #RM, or % #RM (e.g., 75% 1RM, 10RM, or 90% 5RM)	Tschopp et al. [50] Raymond et al. [51] Csapo and Alegre [52] Schoenfeld et al. [53] Schoenfeld et al. [54] Hansen et al. [55] Grgic [56] João et al. [57] Souza et al. [58] Lacio et al. [59] Lopez et al. [60] Refalo et al. [61] Carvalho et al. [62]
	4b	Report the subjective exercise intensity, e.g., the rate of perceived exertion (RPE)	Zhang et al. [63] Hickmott et al. [64]
	4c	If applicable, report the relative training intensity. For example, if a participant performs 5 repetitions of squat with 90 kg and their 5RM is 100 kg, then the relative intensity is 90 kg ÷ 100 kg = 90%	NSCA handbook [3]
Number of repetitions and the set endpoint	5a*	Report the number of repetitions per set	Androulakis-Korakakis et al. [45] Hackett et al. [65]
	5b*	Report whether the participants were asked to train to failure or not	Davies et al. [66] Cerqueira et al. [67] Vieira et al. [68] Grgic et al. [69] Vieira et al. [70]
	5c	Clearly define the set endpoint. For example, the set could be defined to have ended when the participants determined that they could not complete the next repetition (i.e., self-determined repetition maximum). See Steele et al. [27] for detail	Steele et al. [27]
Exercise sequence and the structure of sets	6a	Exercise sequence: Report the sequence of all resistance exercises. If the session included multiple sets, report whether the sequence was traditional (e.g., squat set 1 → squat set 2 → deadlift set 1 → deadlift set 2) or circuit (e.g., squat set 1 → deadlift set 1 → squat set 2 → deadlift set 2). If other training methods were used, such as super-set, complex and contrast training, and multiple-joint to single-joint, report the exercise sequence in detail	Krzysztofik et al. [71] Nunes et al. [72]
	6b	Structure of set: If an alternative set structure (i.e., cluster set and rest redistribution) was used, report the method in detail. Describe the intra-set and inter-repetition rest intervals if applicable	Krzysztofik et al. [71] Latella et al. [73] Jukic et al. [74] Davies et al. [75] Jukic et al. [76]
	6c	Number of sets: Report the number of sets per exercise per training session	Durall et al. [77] Bågenhammar and Hansson [78] Krieger [79] Krieger [80] Ralston et al. [40] Schoenfeld et al. [41] Ralston et al. [81] Androulakis-Korakakis et al. [45] Baz-Valle et al. [46] Baz-Valle et al. [48]
Rest interval between sets and exercises	7*	Report the rest interval between sets and exercises	Grgic et al. [82] Grgic et al. [83] da Silva et al. [84]
Movement tempo	8a*	Report the tempo of resistance exercises in each phase, including but not limited to the durations in eccentric, transition, and concentric phases and the duration between each repetition. If no introduction was provided, state so	Tschopp et al. [50] Schoenfeld et al. [85] Davies et al. [86] Hackett et al. [87] da Rosa Orssatto et al. [88] Krzysztofik et al. [71]
	8b	If applicable, describe how the tempo was set	
Movement velocity	9	Report the movement velocity if recorded	Larsen et al. [89] Zhang et al. [63] Liao et al. [90] Hickmott et al. [64] Orange et al. [91] Zhang et al. [92]
Attentional focus	10*	If applicable, describe the attentional focus strategy, e.g., external focus	Grgic et al. [93] Grgic and Mikulic [94]
Concentric or eccentric-focused training	11a*	If non-traditional resistance training (with both concentric and eccentric phases) was used, report the phase emphasized	Roig et al. [95] Douglas et al. [96] Schoenfeld et al. [97] Molinari et al. [98] Buskard et al. [99] Krzysztofik et al. [71]
	11b	If applicable, describe the method used to emphasize the concentric/eccentric phase of muscle action, e.g., a longer duration of eccentric phase per repetition	
	11c	If the accentuated eccentric loading method was used (i.e., the load was higher during the eccentric phase than during the concentric phase), report the method used to attain the supramaximal eccentric load	
Drop set	12	If applicable, report the number and structure of the drop sets	Krzysztofik et al. [71]
Inter-set intervention	13*	If an inter-set strategy was used (instead of having typical rests between sets), report the method	Latella et al. [100]
**Training program and progression**			
Progression	14a	Describe in detail the decision rule(s) used for determining exercise progression	Adapted from CERT item 7a
	14b	Describe in detail how the exercise program progressed	Adapted from CERT item 7b
Autoregulation method	15	If autoregulation training was used, e.g., autoregulatory progressive resistance exercise, rating of perceived exertion, and velocity-based training programs, report the method	APRE program Larsen et al. [89] Zhang et al. [63]
			RPE program Larsen et al. [89] Zhang et al. [63] Hickmott et al. [64]
			VBT program Larsen et al. [89] Zhang et al. [63] Liao et al. [90] Hickmott et al. [64] Orange et al. [91] Zhang et al. [92]
Programming/ periodization	16	Report the resistance training program (i.e., manipulation of parameters such as intensity and volume). If used, report the model of programming/periodization, e.g., daily undulating and weekly undulating methods	Harries et al. [101] Grgic et al. et al. [102] Williams et al. [103] Grgic et al. [104] Moesgaard et al. [105]
**Exercise setting**			
Equipment	17	Describe in detail the type of exercise equipment used	Adapted from CERT item 1
Location	18	Describe the setting in which the exercises were performed	Adapted from CERT item 12
Supervision	19	Report whether the resistance training was supervised or not. If the resistance training was supervised, report the ratio of instructor to participants	Adapted from CERT item 4
Time	20	Report the time of training (e.g., 10:00–11:00)	Grgic et al. [106]
Blood flow restriction	21a	If blood flow restriction was used, describe the pressure modality: 1. Intermittent or continuous (minutes) 2. Pressure (#mm Hg or % of arterial occlusion pressure) 3. Cuff width (cm) 4. Where the pressure was applied 5. The inflator device	Domingos and Polito [107] Lixandrão et al. [108] Krzysztofik et al. [71] Centner and Lauber [109] Cuyul-Vásquez et al. [110] Ferlito et al. [111] Grønfeldt et al. [112] Cerqueira et al. [113] Cerqueira et al. [67] de Queiros et al. [114] Liu et al. [115] Nitzsche et al. [116] Rodrigo-Mallorca et al. [117] Koc et al. [118]
	21b	If blood flow restriction was used, report how, when, and at what position the arterial occlusion pressure was determined	
Elastic resistance training	22	If applicable, report the method and equipment used	Lopes et al. [119]
Inertial resistance training	23	If applicable, report the method and equipment used	Vicens-Bordas et al. [120]
Training in hypoxia	24	If applicable, report the setting in which the training occurred	Ramos-Campo et al. [121]
**Planned vs. actual training**			
Adherence	25	Describe how adherence or fidelity to the exercise intervention was assessed/measured, e.g., via drop-out rate and attendance rate	Adapted from CERT 16a
Compliance and deviation	26	Describe the extent to which the intervention was delivered as planned. Report the deviation from the original exercise protocol	Adapted from CERT 16b

CERT: Consensus on Exercise Reporting Template. NSCA: National Strength and Conditioning Association. *: These subitems were also included in the checklist by Coratella 2022 [23] as “within-exercise variables”

## Discussion

The Preferred Reporting Items for Resistance Exercise Studies (PRIRES) checklist comprises 26 reporting items that cover four major topics of research on resistance exercise training: 1) exercise selection, performance, and training parameters, 2) training program and progression, 3) exercise setting, and 4) planned vs actual training.

The first section of this reporting checklist, exercise selection, performance, and training parameters, covers the content and flow of a given resistance exercise session. The second section, training program and progression, covers the rules and decisions related to the training progression and to the methods specific to resistance training, such as velocity-based training and periodization. The third section of the checklist, exercise setting, reminds users to report the environment in which the resistance exercise was conducted. Planned vs actual training, the fourth section, points out that researchers should provide information about how well the participants were able to adhere to the intervention plan and about unintended deviations that occurred.

Two subitems of the PRIRES checklist, namely those relating to relative intensity (Item 4c) and set endpoint (Item 5c), were neither identified from the included systematic reviews nor adapted from the CERT checklist. Retrieved from an NSCA handbook [3] (T15.17), Item 4c addresses relative training intensity, which, unlike absolute training intensity, reflects not only the absolute load but also the number of repetitions in a set. Therefore, it can more accurately indicate how difficult a given set is. Item 5c, set endpoint, is adapted from an article by Steele et al. [27]. In this article, they showed the ambiguity in the use and definition of the set endpoint [27(Pg368-370)] and provided recommendations [27(T3)] on how to report it. Both relative intensity and a clearly defined set endpoint are essential measures for comparing resistance exercise studies.

The PRIRES checklist was designed specifically for reporting resistance exercise interventions and is expected to be used with other reporting guidelines. For example, for a resistance training RCT, PRIRES can be used with CONSORT [28] which covers the overall design of a trial, such as the randomization process and masking (i.e., blinding). PRIRES can also be applied to an interventional study protocol and used with the Standard Protocol Items: Recommendations for Interventional Trials (SPIRIT) [29]. The editable checklists for both completed studies and protocol are provided in Additional files 6 and 7.

The article published in 2022 by Coratella [23] provided a comprehensive view of how training variables beyond load and number of repetitions influence training stimuli. For instance, Coratella explained that even when the load, number of repetitions, and displacement per repetition are fixed, the differences in muscle length being trained can affect acute as well as long-term results [23 (PP. 4–5)]. Moreover, Coratella also provided in-depth coverage of the influences of manipulations of these training variables on muscle strength and hypertrophy. In the PRIRES checklist, we address the training volume issue by listing the volume-related variables individually instead of labeling a single “training volume” item and consider the examination of the training effect of reporting items as out of scope when developing a checklist. Investigating the training effect of these items in a future umbrella review is expected to be valuable [30].

This article presents the development process and resulting items of the PRIRES checklist but not why each item is critical and relevant. Motivated by the CONSORT 2010 explanation and elaboration [31], we are preparing an accompanying article [32], which will describe the rationale, importance, and examples regarding each item.

***In-text citation***: This article follows the more precise citation method [33]. In-text citation: I: introduction; M: method; R: results; D: discussion; C: conclusion; the number after abbreviation: paragraph (para.); T: table; F: figure; Sec: section; Pg: page.

### Registration

This study is registered with the EQUATOR Network and is available at https://www.equator-network.org/library/reporting-guidelines-under-development/reporting-guidelines-under-development-for-clinical-trials/#PRIRES. The protocol is registered with PROSPERO (CRD42021235259).

## Conclusions

The PRIRES checklist was developed primarily via carrying out an umbrella review of systematic reviews and adapting the existing CERT checklist. This development process allowed PRIRES to address the research questions that have been systematically studied. The checklist is expected to improve the reporting completeness of future resistance exercise studies.

### Supplementary Information

**Additional file 1**. Original Rationale and Objective.

**Additional file 2**. Umbrella Review Reporting Checklist.

**Additional file 3**. Syntax of Literature Search.

**Additional file 4**. Records Excluded After the Full-Text Review.

**Additional file 5**. Included Reviews and Research Questions.

**Additional file 6**. Preferred Reporting Items for Resistance Exercise Studies (PRIRES) Checklist for Completed Studies.

**Additional file 7**. Preferred Reporting Items for Resistance Exercise Studies (PRIRES) Checklist for Protocols.

## Data Availability

All available data were presented in this manuscript and supplementary information.

## Abbreviations

PRIRES: Preferred Reporting Items for Resistance Exercise Studies
EQUATOR: Enhancing the QUAlity and Transparency Of health Research
PROSPERO: International prospective register of systematic reviews
CERT: Consensus on Exercise Reporting Template
NSCA: National Strength and Conditioning Association
CONSORT: Consolidated Standards of Reporting Trials
SPIRIT: Standard Protocol Items: Recommendations for Interventional Trials
RCT: Randomized controlled trial
