# Supplementary material for: Preferred Reporting Items for Resistance Exercise Studies (PRIRES): A Checklist Developed Using an Umbrella Review of Systematic Reviews
Source: Sports Med Open. 2023 Dec 1;9:114. doi: 10.1186/s40798-023-00640-1 (PMC10692055; doi:10.1186/s40798-023-00640-1)
Supplement: Supplementary file 1 — Additional file 1. Original Rationale and Objective. [file 40798_2023_640_MOESM1_ESM.docx]

# Additional File 1: Original Rationale and Objective

The issues of replication and scientific transparency have been raised in many research fields including psychology [1(T1)], social science [2(Pg638)], and medicine [3(T1)]. Problems pertaining to reliability have also been raised in exercise and sports science research [4, 5]. Potential means to address the replication crisis and enhance research reliability include trial registration [6(Sec3.2)], publishing the protocol before data collection, the registered reports) [6(Sec3.3), 7], a results-free peer review [8(Pg6-7)], increasing sample sizes [5, 9], conducting replication studies [1, 2, 4(Pg130)], and improving transparency [4(Pg130-131)] and reporting completeness [10].

Although the Consolidated Standards of Reporting Trials (CONSORT)[10, 11] and Consensus on Exercise Reporting Template (CERT)[12] have been published to enhance the reporting quality of randomized controlled trials (RCTs) and exercise interventional studies, respectively, a supplementary preferred reporting items checklist can further improve the reporting quality of resistance exercise studies. For instance, there were concerns regarding the reported resistance exercise method and program of 7 of the 11 studies summarized in our previous studies [13(I3, T1)]. Specifically, of these 7 studies, three failed to report basic items related to resistance exercise, i.e. repetition, intensity, and rest intervals. In addition, some items regarding the resistance exercise such as the rest interval between sets and exercises and the order of exercises, which had been reported in our previous studies [13(F2)], are not fully covered by the CONSORT [10, 11] and CERT [12] checklists. Thus, a supplementary reporting checklist for resistance exercise studies can be beneficial to future research.

To overcome the limitations of the Delphi technique, which has been used to develop existing reporting checklists such as CONSORT [14(M)], an umbrella review will be applied in this study. The Delphi technique was first proposed by Norman Dalkey and Olaf Helmer in the 1950s to develop consensus among experts [15(I3)]. Although it can provide some information, it suffers from several methodological disadvantages that can be avoided using the newer technique of umbrella review. An umbrella review is a tertiary research design (in contrast to primary research such as RCTs and secondary research such as systematic reviews)[16(Pg5-6)] that emerged at the beginning of the 21^st^ century. This design enables systematic data collection and synthesis on a broad issue, which is impractical for a traditional systematic review. Following are several major comparisons between these two research methods that are of consequence to our study. First, expert opinions in the Delphi technique are ranked the lowest in the evidence hierarchy, as opposed to the umbrella review wherein they are considered the highest [16(F2.1)]. Second, concerns have been raised that the Delphi technique is not fully “systematic,” which leads to consequent problems. For instance, Humphrey-Murto and de Wit criticized the Delphi method for the ambiguity of its methodology, poor reporting quality, and the presence of little to no empirical evidence to support best practices in the consensus development stages [17(Pg136)]. Third, regarding the advantages of the proposed checklist, as a supplement to CONSORT it will need to be updated regularly and timely for it to function optimally. The feasibility of rapidly developing and updating an umbrella review will be an advantage over the time-consuming Delphi technique. A detailed discussion of the pros and cons of the Delphi technique is beyond the scope of this protocol. An integrative introduction to the umbrella review has been edited by Biondi-Zoccai [16]. The limitations of the Delphi technique in methodology, process, results, and conclusion have been reviewed by Vernon [18], and the disadvantages of this technique, including researcher bias and shortcomings, unethical behavior caused by anonymity, and debates over the method rather than the topic, have been discussed by Avella [19].

This study aims to construct a reporting checklist as a supplement to the existing reporting guidelines such as CONSORT [10, 11], specifically for resistance exercise studies. A preferred reporting items checklist developed using umbrella review methods promises to be more systematic and provide a higher level of evidence than those developed using the Delphi technique.

# References

1. Open Science Collaboration. PSYCHOLOGY. Estimating the reproducibility of psychological science. Science. 2015;349(6251):aac4716.

2. Camerer CF, Dreber A, Holzmeister F, Ho T-H, Huber J, Johannesson M, et al. Evaluating the replicability of social science experiments in Nature and Science between 2010 and 2015. Nature Human Behaviour. 2018;2(9):637-44.

3. Errington TM, Mathur M, Soderberg CK, Denis A, Perfito N, Iorns E, et al. Investigating the replicability of preclinical cancer biology. eLife. 2021;10:e71601.

4. Halperin I, Vigotsky AD, Foster C, Pyne DB. Strengthening the Practice of Exercise and Sport-Science Research. Int J Sports Physiol Perform. 2018;13(2):127-34.

5. Schweizer G, Furley P. Reproducible research in sport and exercise psychology: The role of sample sizes. Psychology of Sport and Exercise. 2016;23:114-22.

6. Caldwell AR, Vigotsky AD, Tenan MS, Radel R, Mellor DT, Kreutzer A, et al. Moving Sport and Exercise Science Forward: A Call for the Adoption of More Transparent Research Practices. Sports Medicine. 2020;50(3):449-59.

7. Law LS-C, Lo EA-G. A two-stage review process for randomized controlled trials: the ultimate solution for publication bias? Canadian Journal of Anesthesia/Journal canadien d'anesthésie. 2016;63(12):1381-2.

8. Button KS, Bal L, Clark A, Shipley T. Preventing the ends from justifying the means: withholding results to address publication bias in peer-review. BMC Psychology. 2016;4(1):59.

9. Button KS, Ioannidis JPA, Mokrysz C, Nosek BA, Flint J, Robinson ESJ, et al. Power failure: why small sample size undermines the reliability of neuroscience. Nature Reviews Neuroscience. 2013;14(5):365-76.

10. Moher D, Hopewell S, Schulz KF, Montori V, Gøtzsche PC, Devereaux PJ, et al. CONSORT 2010 Explanation and Elaboration: updated guidelines for reporting parallel group randomised trials. BMJ. 2010;340:c869.

11. Dwan K, Li T, Altman DG, Elbourne D. CONSORT 2010 statement: extension to randomised crossover trials. BMJ. 2019;366:l4378.

12. Slade SC, Dionne CE, Underwood M, Buchbinder R. Consensus on Exercise Reporting Template (CERT): Explanation and Elaboration Statement. British Journal of Sports Medicine. 2016;50(23):1428-37.

13. Lin T-Y, Hsieh S-S, Chueh T-Y, Huang C-J, Hung T-M. The effects of barbell resistance exercise on information processing speed and conflict-related ERP in older adults: a crossover randomized controlled trial. Scientific Reports. 2021;11(1):9137.

14. Begg C, Cho M, Eastwood S, Horton R, Moher D, Olkin I, et al. Improving the quality of reporting of randomized controlled trials. The CONSORT statement. Jama. 1996;276(8):637-9.

15. Barrett D, Heale R. What are Delphi studies? Evidence Based Nursing. 2020;23(3):68-9.

16. Biondi-Zoccai G. Umbrella Reviews: Evidence Synthesis with Overviews of Reviews and Meta-epidemiologic Studies: Springer; 2016.

17. Humphrey-Murto S, de Wit M. The Delphi method-more research please. J Clin Epidemiol. 2019;106:136-9.

18. Vernon W. The Delphi technique: A review. International Journal of Therapy and Rehabilitation. 2009;16(2):69-76.

19. Avella J. Delphi Panels: Research Design, Procedures, Advantages, and Challenges. International Journal of Doctoral Studies. 2016;11:305-21.
