# Supplementary material for: Preferred Reporting Items for Resistance Exercise Studies (PRIRES): A Checklist Developed Using an Umbrella Review of Systematic Reviews
Source: Sports Med Open. 2023 Dec 1;9:114. doi: 10.1186/s40798-023-00640-1 (PMC10692055; doi:10.1186/s40798-023-00640-1)
Supplement: Supplementary file 3 — Additional file 3. Syntax of Literature Search. [file 40798_2023_640_MOESM3_ESM.docx]

# Additional File 3: Syntax of Literature Search

## Scopus:

[TITLE-ABS (“resistance exercise*” OR “resistance train*” OR “weight exercise*” OR “weight train*” OR “weight bear*” OR “weight-bear*” OR weightlift* OR “weight lift*” OR “strength train*” OR “strength exercise*” OR “power train*” OR “power exercise” OR “explosive exercise*”) AND TITLE (systematic OR meta)]

## PubMed:

(“resistance exercise*” [Title/Abstract] OR “resistance train*” [Title/Abstract] OR “weight exercise*” [Title/Abstract] OR “weight train*” [Title/Abstract] OR “weight bear*” [Title/Abstract] OR “weight-bear*” [Title/Abstract] OR weightlift* [Title/Abstract] OR weight lift* [Title/Abstract] OR “strength train*” [Title/Abstract] OR “strength exercise*” [Title/Abstract] OR “power train*” [Title/Abstract] OR “power exercise” [Title/Abstract] OR “explosive exercise*” [Title/Abstract]) AND (systematic [Title] OR meta [Title])
