# Supplementary material for: Preferred Reporting Items for Resistance Exercise Studies (PRIRES): A Checklist Developed Using an Umbrella Review of Systematic Reviews
Source: Sports Med Open. 2023 Dec 1;9:114. doi: 10.1186/s40798-023-00640-1 (PMC10692055; doi:10.1186/s40798-023-00640-1)
Supplement: Supplementary file 4 — Additional file 4. Records Excluded After the Full-Text Review. [file 40798_2023_640_MOESM4_ESM.docx]

# Additional File 4: Records Excluded After the Full-Text Review

| Reason 1: Included studies that did not directly compare different resistance training methods or included non-resistance exercise intervention.  Reason 2: Not a systematic review.  Reason 3: Not published in English.  Reason 4: Retraction.  Reason 5: Included non-interventional studies. | |
| --- | --- |
| Study | Reason for exclusion |
| Rhea and Alderman (2004)  [1] | Reason 1 (References). |
| Neto et al. (2009)  [2] | Reason 3. |
| Steib et al. (2010)  [3] | Reason 1 (Table 2). |
| Nicola and Catherine (2011)  [4] | Reason 2 (Abstract). |
| Behm et al. (2015)  [5] | Reason 1 (Table 1). |
| Soria-Gila et al. (2015)  [6] | Reason 4 [7, 8]. |
| Behm et al. (2017)  [9] | Reason 1 (Table 2). |
| Cirer-Sastre et al. (2018)  [10] | Reason 1 (Table 1). |
| Afonso et al. (2019)  [11] | Reason 2 (“Eligibility Criteria”). |
| Centner et al. (2019)  [12] | Reason 1 (Section 2.2 para. 1). |
| Katsoulis et al. (2019)  [13] | Reason 1 (“Eligibility Criteria”). |
| Acosta-Manzano et al. (2020)  [14] | Reason 1 (Table 1). |
| Cormier et al. (2020)  [15] | Reason 1 (Table 1). |
| Naunton et al. (2020)  [16] | Reason 1 (Table 1). |
| Thompson et al. (2020)  [17] | Reason 1 (Table 1). |
| Barahona-Fuentes et al. (2021)  [18] | Reason 1 (Section 2.1). |
| Cormier et al. (2021)  [19] | Reason 1 (“Eligibility criteria” para. 2). |
| Garbisu-Hualde and Santos-Concejero (2021)  [20] | Reason 1 (“Inclusion criteria”). |
| Grantham et al. (2021)  [21] | Reason 1 (Section 2.3 para. 2). |
| Krzysztofik et al. (2021)  [22] | Reason 1 (“Inclusion and Exclusion Criteria” para. 2). |
| Lopez, Galvão, et al. (2021)  [23] | Reason 1 (“Study selection procedure”). |
| Lopez, Taaffe, Newton, Buffart, et al. (2021)  [24] | Reason 1 (“Study selection procedure” para. 2). |
| Lopez, Taaffe, Newton and Galvão (2021)  [25] | Reason 1 (“Study selection procedure” para. 2). |
| Manojlović et al. (2021)  [26] | Reason 1 (“Inclusion and Exclusion Criteria”). |
| Marshall et al. (2021)  [27] | Reason 1 (Table 3). |
| Polito, Dias, et al. (2021)  [28] | Reason 1 (“Eligibility Criteria”). |
| Polito, Papst, et al. (2021)  [29] | Reason 1 (“Search strategy and selection criteria” para. 2). |
| Ruku et al. (2021)  [30] | Reason 1 (“Data sources and systematic literature review” para. 1). |
| Sahabuddin et al. (2021)  [31] | Reason 1 (Table 1). |
| Santos et al. (2021)  [32] | Reason 1 (“Eligibility criteria”). |
| Uthoff et al. (2021)  [33] | Reason 1 (Table 2). |
| Yang et al. (2021)  [34] | Reason 1 (“Study Selection”). |
| Coelho-Júnior et al. (2022)  [35] | Reason 1 (Section 2.2.1). |
| Čretnik et al. (2022)  [36] | Reason 1 (Table 1). |
| da Silva et al. (2022)  [37] | Reason 1 (“Type of intervention investigated in the studies”). |
| Fone and van den Tillaar (2022)  [38] | Reason 1 (Table 1). |
| Guo et al. (2022)  [39] | Reason 1 (“Inclusion and Exclusion Criteria”). |
| Hashmi et al. (2022)  [40] | Reason 1 (Table 1). |
| Igarashi (2022)  [41] | Reason 1 (“Study selection”). |
| Kulkarni et al. (2022)  [42] | Reason 1 (Table 2). |
| Labata-Lezaun et al. (2022)  [43] | Reason 1 (Table 1). |
| Li et al. (2022)  [44] | Reason 1 (Table 1). |
| Liao et al. (2022)  [45] | Reason 1 (“Studies’ Characteristics” para. 2). |
| Liu et al. (2022)  [46] | Reason 1 (“Inclusion Criteria”). |
| Maurits Ruku et al. (2022)  [47] | Reason 1 (Table 1). |
| Muniz-Pardos et al. (2022)  [48] | Reason 5 (Figure 1). |
| Sinclair et al. (2022)  [49] | Reason 1 (References). |
| Wang et al. (2022)  [50] | Reason 1 (“Inclusion and exclusion criteria”). |
| Zhang et al. (2022)  [51] | Reason 1 (Table 1). |

## References

1. Rhea MR, Alderman BL. A meta-analysis of periodized versus nonperiodized strength and power training programs. Res Q Exerc Sport. 2004;75(4):413-22.

2. Neto AGC, da Silva NL, Farinatti PTV. Influence of resistance training variables on post-exercise oxygen consumption: A systematic review. Rev Bras Med Esporte. 2009;15(1):70-8.

3. Steib S, Schoene D, Pfeifer K. Dose-response relationship of resistance training in older adults: A meta-analysis. Medicine and Science in Sports and Exercise. 2010;42(5):902-14.

4. Nicola F, Catherine S. Dose-response relationship of resistance training in older adults: A meta-analysis. Br J Sports Med. 2011;45(3):233-4.

5. Behm DG, Muehlbauer T, Kibele A, Granacher U. Effects of Strength Training Using Unstable Surfaces on Strength, Power and Balance Performance Across the Lifespan: A Systematic Review and Meta-analysis. Sports Med. 2015;45(12):1645-69.

6. Soria-Gila MA, Chirosa IJ, Bautista IJ, Baena S, Chirosa LJ. Effects of variable resistance training on maximal strength: A meta-analysis. J Strength Cond Res. 2015;29(11):3260-70.

7. Nilo Dos Santos WD, Gentil P, Lima de Araújo Ribeiro A, Vieira CA, Martins WR. Effects of Variable Resistance Training on Maximal Strength: A Meta-analysis. J Strength Cond Res. 2018;32(11):e52-e5.

8. Effects of Variable Resistance Training on Maximal Strength: A Meta-Analysis: Retraction. J Strength Cond Res. 2018;32(11):e56.

9. Behm DG, Young JD, Whitten JHD, Reid JC, Quigley PJ, Low J, et al. Effectiveness of traditional strength vs. power training on muscle strength, power and speed with youth: A systematic review and meta-analysis. Front Physiol. 2017;8(JUN).

10. Cirer-Sastre R, Beltrán-Garrido JV, Corbi F. Contralateral effects after unilateral strength training: A meta-analysis comparing training loads. J Sports Sci Med. 2018;17(1):163-6.

11. Afonso J, Rocha T, Nikolaidis PT, Clemente FM, Rosemann T, Knechtle B. A systematic review of meta-analyses comparing periodized and non-periodized exercise programs: Why we should go back to original research. Front Physiol. 2019;10(AUG).

12. Centner C, Wiegel P, Gollhofer A, König D. Effects of Blood Flow Restriction Training on Muscular Strength and Hypertrophy in Older Individuals: A Systematic Review and Meta-Analysis. Sports Med. 2019;49(1):95-108.

13. Katsoulis K, Stathokostas L, Amara CE. The effects of high- versus low-intensity power training on muscle power outcomes in healthy, older adults: A systematic review. J Aging Phys Act. 2019;27(3):422-39.

14. Acosta-Manzano P, Rodriguez-Ayllon M, Acosta FM, Niederseer D, Niebauer J. Beyond general resistance training. Hypertrophy versus muscular endurance training as therapeutic interventions in adults with type 2 diabetes mellitus: A systematic review and meta-analysis. Obes Rev. 2020;21(6).

15. Cormier P, Freitas TT, Rubio-Arias JÁ, Alcaraz PE. Complex and Contrast Training: Does Strength and Power Training Sequence Affect Performance-Based Adaptations in Team Sports? A Systematic Review and Meta-analysis. J Strength Cond Res. 2020;34(5):1461-79.

16. Naunton J, Street G, Littlewood C, Haines T, Malliaras P. Effectiveness of progressive and resisted and non-progressive or non-resisted exercise in rotator cuff related shoulder pain: a systematic review and meta-analysis of randomized controlled trials. Clin Rehabil. 2020;34(9):1198-216.

17. Thompson SW, Rogerson D, Ruddock A, Barnes A. The Effectiveness of Two Methods of Prescribing Load on Maximal Strength Development: A Systematic Review. Sports Med. 2020;50(5):919-38.

18. Barahona-Fuentes G, Huerta Ojeda Á, Chirosa-Ríos L. Effects of Training with Different Modes of Strength Intervention on Psychosocial Disorders in Adolescents: A Systematic Review and Meta-Analysis. Int J Environ Res Public Health. 2021;18(18).

19. Cormier P, Freitas TT, Seaman K. A systematic review of resistance training methodologies for the development of lower body concentric mean power, peak power, and mean propulsive power in team-sport athletes. Sports Biomech. 2021.

20. Garbisu-Hualde A, Santos-Concejero J. Post-Activation Potentiation in Strength Training: A Systematic Review of the Scientific Literature. J Hum Kinet. 2021;78(1):141-50.

21. Grantham B, Korakakis V, O'Sullivan K. Does blood flow restriction training enhance clinical outcomes in knee osteoarthritis: A systematic review and meta-analysis. Phys Ther Sport. 2021;49:37-49.

22. Krzysztofik M, Wilk M, Stastny P, Golas A. Post-activation Performance Enhancement in the Bench Press Throw: A Systematic Review and Meta-Analysis. Front Physiol. 2021;11.

23. Lopez P, Galvão DA, Taaffe DR, Newton RU, Souza G, Trajano GS, et al. Resistance training in breast cancer patients undergoing primary treatment: a systematic review and meta-regression of exercise dosage. Breast Cancer. 2021;28(1):16-24.

24. Lopez P, Taaffe DR, Newton RU, Buffart LM, Galvão DA. What is the minimal dose for resistance exercise effectiveness in prostate cancer patients? Systematic review and meta-analysis on patient-reported outcomes. Prostate Cancer Prostatic Dis. 2021;24(2):465-81.

25. Lopez P, Taaffe DR, Newton RU, Galvão DA. Resistance Exercise Dosage in Men with Prostate Cancer: Systematic Review, Meta-analysis, and Meta-regression. Medicine and science in sports and exercise. 2021;53(3):459-69.

26. Manojlović D, Kozinc Ž, Šarabon N. Trunk, hip and knee exercise programs for pain relief, functional performance and muscle strength in patellofemoral pain: Systematic review and meta-analysis. J Pain Res. 2021;14:1431-49.

27. Marshall J, Bishop C, Turner A, Haff GG. Optimal Training Sequences to Develop Lower Body Force, Velocity, Power, and Jump Height: A Systematic Review with Meta-Analysis. Sports Med. 2021;51(6):1245-71.

28. Polito MD, Dias JR, Jr., Papst RR. Resistance training to reduce resting blood pressure and increase muscle strength in users and non-users of anti-hypertensive medication: A meta-analysis. Clin Exp Hypertens. 2021;43(5):474-85.

29. Polito MD, Papst RR, Farinatti P. Moderators of strength gains and hypertrophy in resistance training: A systematic review and meta-analysis. J Sports Sci. 2021;39(19):2189-98.

30. Ruku DM, Tran Thi TH, Chen HM. Effect of center-based or home-based resistance training on muscle strength and VO(2) peak in patients with HFrEF: A systematic review and meta-analysis. Enferm Clin (Engl Ed). 2021.

31. Sahabuddin FNA, Jamaludin NI, Amir NH, Shaharudin S. The effects of hip- and ankle-focused exercise intervention on dynamic knee valgus: a systematic review. PeerJ. 2021;9:e11731.

32. Santos CS, Pinto JR, Scoz RD, Alves BM, Oliveira PR, Soares WJ, et al. What is the traditional method of resistance training: a systematic review. J Sports Med Phys Fitness. 2021.

33. Uthoff A, Sommerfield LM, Pichardo AW. Effects of resistance training methods on golf clubhead speed and hitting distance: A systematic review. J Strength Cond Res. 2021;35(9):2651-60.

34. Yang Y, Chen SC, Chen CN, Hsu CW, Zhou WS, Chien KY. Training Session and Detraining Duration Affect Lower Limb Muscle Strength Maintenance in Middle-Aged and Older Adults: A Systematic Review and Meta-Analysis. J Aging Phys Act. 2021:1-15.

35. Coelho-Júnior HJ, Silva Aguiar S, Calvani R, Picca A, de Azevedo Carvalho D, Rodrigues B, et al. Acute and chronic effects of traditional and high-speed resistance training on blood pressure in older adults: A crossover study and systematic review and meta-analysis. Exp Gerontol. 2022;163.

36. Čretnik K, Pleša J, Kozinc Ž, Löfler S, Šarabon N. The Effect of Eccentric vs. Traditional Resistance Exercise on Muscle Strength, Body Composition, and Functional Performance in Older Adults: A Systematic Review With Meta-Analysis. Front Sports Act Living. 2022;4:873718.

37. da Silva JM, de Barros BS, Almeida GJ, O’Neil J, Imoto AM. Dosage of resistance exercises in fibromyalgia: evidence synthesis for a systematic literature review up-date and meta-analysis. Rheumatol Int. 2022;42(3):413-29.

38. Fone L, van den Tillaar R. Effect of Different Types of Strength Training on Swimming Performance in Competitive Swimmers: A Systematic Review. Sports Med - Open. 2022;8(1).

39. Guo W, Soh KG, Zakaria NS, Hidayat Baharuldin MT, Gao Y. Effect of Resistance Training Methods and Intensity on the Adolescent Swimmer's Performance: A Systematic Review. Front Public Health. 2022;10.

40. Hashmi MA, Kazmi SAM, Ali S. Impact of Resistance Training on FEV1 and Functional Exercise Capacity among COPD Patients: A Meta-analysis. J Coll Phys Surg Pak. 2022;32(1):68-74.

41. Igarashi Y. Effects of Differences in Exercise Programs With Regular Resistance Training on Resting Blood Pressure in Hypertensive Adults: A Systematic Review and Meta-Analysis. J Strength Cond Res. 2022.

42. Kulkarni D, Gregory S, Evans M. Effectiveness of eccentric-biased exercise interventions in reducing the incidence of falls and improving functional performance in older adults: a systematic review. Eur Geriatr Med. 2022;13(2):367-80.

43. Labata-Lezaun N, Llurda-Almuzara L, González-Rueda V, López-de-Celis C, Cedeño-Bermúdez S, Bañuelos-Pago J, et al. Effectiveness of Blood Flow Restriction Training on Muscle Strength and Physical Performance in Older Adults: A Systematic Review and Meta-analysis. Arch Phys Med Rehabil. 2022.

44. Li S, Li S, Wang L, Quan H, Yu W, Li T, et al. The Effect of Blood Flow Restriction Exercise on Angiogenesis-Related Factors in Skeletal Muscle Among Healthy Adults: A Systematic Review and Meta-Analysis. Front Physiol. 2022;13.

45. Liao KF, Nassis GP, Bishop C, Yang W, Bian C, Li YM. Effects of unilateral vs. bilateral resistance training interventions on measures of strength, jump, linear and change of direction speed: a systematic review and meta-analysis. Biol Sport. 2022;39(3):485-97.

46. Liu X, Gao Y, Lu J, Ma Q, Shi Y, Liu J, et al. Effects of Different Resistance Exercise Forms on Body Composition and Muscle Strength in Overweight and/or Obese Individuals: A Systematic Review and Meta-Analysis. Front Physiol. 2022;12.

47. Maurits Ruku D, Tran Thi TH, Chen HM. Effect of center-based or home-based resistance training on muscle strength and VO2 peak in patients with Heart Failure: A systematic review and meta-analysis. Enferm Clin. 2022;32(2):103-14.

48. Muniz-Pardos B, Gomez-Bruton A, Matute-Llorente A, Gonzalez-Aguero A, Gomez-Cabello A, Gonzalo-Skok O, et al. Nonspecific Resistance Training and Swimming Performance: Strength or Power? A Systematic Review. J Strength Cond Res. 2022;36(4):1162-70.

49. Sinclair P, Kadhum M, Paton B. Tolerance to Intermittent vs Continuous Blood Flow Restriction Training: A meta-Analysis. Int J Sports Med. 2022;43(1):3-10.

50. Wang HN, Chen Y, Cheng L, Cai YH, Li W, Ni GX. Efficacy and Safety of Blood Flow Restriction Training in Patients With Knee Osteoarthritis: A Systematic Review and Meta-Analysis. Arthritis Care Res. 2022;74(1):89-98.

51. Zhang T, Wang X, Wang J. Effect of blood flow restriction combined with low-intensity training on the lower limbs muscle strength and function in older adults: A meta-analysis. Exp Gerontol. 2022:111827.
