# Supplementary material for: Preferred Reporting Items for Resistance Exercise Studies (PRIRES): A Checklist Developed Using an Umbrella Review of Systematic Reviews
Source: Sports Med Open. 2023 Dec 1;9:114. doi: 10.1186/s40798-023-00640-1 (PMC10692055; doi:10.1186/s40798-023-00640-1)
Supplement: Supplementary file 5 — Additional file 5. Included Reviews and Research Questions. [file 40798_2023_640_MOESM5_ESM.docx]

# Additional File 5: Included Reviews and Research Questions

| Study | Research questions (quotations) |
| --- | --- |
| Durall et al. (2006)  [1] | This systematic review of randomized controlled trials (RCTs) comparing strength gains following single-set (SS) and multiple-set (MS) resistance-training protocols was performed to formulate therapeutic exercise recommendations. (Abstract) |
| Bågenhammar and Hansson (2007)  [2] | [T]he aim of this study was to review studies concerning single-set and multiple-set resistance training. (p. 155) |
| Krieger (2009)  [3] | The purpose of this article was to improve upon the limitations of previous meta-analyses and use hierarchical, random-effects meta-regression to compare the effects of single and multiple sets per exercise on dynamic strength. A second purpose was to establish a dose-response effect of set volume on strength. The hypothesis was that multiple sets would improve strength to a greater degree than single sets. (p. 1891) |
| Roig et al. (2009)  [4] | The aim of this systematic review was to determine if eccentric exercise is superior to concentric exercise in stimulating gains in muscle strength and mass. (Abstract) |
| Krieger (2010)  [5] | The purpose of this paper was to use meta-analysis to compare the effects of single and multiple sets per exercise on muscle hypertrophy. A second purpose was to establish a dose–response effect of set volume on hypertrophy. (p. 1151) |
| Tschopp et al. (2011)  [6] | Objective: to determine the effects of power training with high movement velocity compared with conventional resistance training with low movement velocity for older community-dwelling people. (Abstract) |
| Raymond et al. (2013)  [7] | The primary aims were to examine the effectiveness of HIPRST [high intensity progressive resistance strength training] in older adults in improving strength, endurance, and functional performance and assess its safety compared with other intensities of PRST. Secondary aims were to examine the effect of HIPRST on cognition, psychological status, QOL [quality of life], falls rate, power, flexibility, and cardiovascular fitness in those who have undertaken such training. (p. 1459) |
| Harries et al. (2015)  [8] | The aims of this review were to (a) systematically identify and examine all studies directly comparing linear and undulating periodized RT programs and to synthesize the results, (b) quantitatively compare linear and undulating periodized RT programs’ effects on muscular strength using meta-analysis, (c) evaluate the risk of bias in previous studies and provide recommendations to improve the quality of future studies, and (d) review the study populations in which the comparisons of these resistance training programs have been investigated.  (Introduction para. 3) |
| Schoenfeld et al. (2015)  [9] | The purpose of this paper, therefore, is to conduct a systematic review and meta-analysis as to the effects of repetition duration on muscle growth in an effort to provide clarity on the topic. (Introduction para. 4) |
| Csapo and Alegre (2016)  [10] | The goals of the present manuscript are, therefore, to perform a meticulous review of articles comparing the efficacy of heavy and light-moderate load RT [resistance training] as opposed to no exercise in elderly cohorts.  (Introduction para. 4) |
| Davies et al. (2016)  [11] | We conducted a systematic review and meta-analysis to examine the effect of failure versus non-failure training on muscular strength.  (Abstract) |
| Schoenfeld, Ogborn, et al. (2016)  [12] | The purpose of this paper therefore is threefold: (1) to systematically and objectively review the literature that directly investigates the effects of RT frequency on muscle hypertrophy; (2) to quantify these effects via meta-analyses; and (3) to draw evidence-based conclusions on the topic to guide exercise program design.  (Introduction para. 4) |
| Schoenfeld, Wilson, et al. (2016)  [13] | The purpose of this paper therefore was to conduct a meta-analysis to compare muscular adaptations between low- and high-load resistance training programmes.  (Introduction para. 4) |
| Davies et al. (2017)  [14] | We conducted a systematic review and meta-analysis to examine the effect of movement velocity during resistance training on dynamic muscular strength.  (Abstract) |
| Douglas et al. (2017)  [15] | The purpose of this systematic review was to determine the effects of eccentric training in comparison to concentric-only or traditional (i.e. constrained by concentric strength) resistance training.  (Abstract) |
| Grgic, Lazinica, et al. (2017)  [16] | Accordingly, the purpose of this paper was to systematically review the literature and objectively assess the effects of short versus long inter-set rest intervals in resistance training and their impact on long-term muscle hypertrophy.  (Introduction para. 5) |
| Grgic, Mikulic, et al. (2017)  [17] | To provide a more in-depth look at the topic, we undertook a systematic review of the literature and a meta-analysis of intervention trials comparing the effects of linear periodization (LP) and daily undulating periodization (DUP) resistance training programs on muscle hypertrophy. (Abstract) |
| Ralston et al. (2017)  [18] | We conducted a meta-analysis with restrictive inclusion criteria and examined the potential effects of low (LWS), medium (MWS) or high weekly set (HWS) strength training on muscular strength per exercise. Secondly, we examined strength gain variations when performing multi-joint or isolation exercises, and probed for a potential relationship between weekly set number and stage of subjects’ training (trained versus untrained).  (Abstract) |
| Schoenfeld, Grgic, et al. (2017)  [19] | [T]he purpose of this article was to conduct a systematic review of the current body of literature and a meta-analysis to compare changes in strength and hypertrophy between low vs. high-load RT protocols.  (Introduction para. 4) |
| Schoenfeld, Ogborn and Krieger (2017)  [20] | The purpose of this paper was to systematically review the current literature and elucidate the effects of total weekly resistance training (RT) volume on changes in measures of muscle mass via meta-regression.  (Abstract) |
| Schoenfeld, Ogborn, Vigotsky, et al. (2017)  [21] | Given the gaps in our knowledge base, the purpose of this article was to systematically review the current literature in an effort to elucidate the hypertrophic effects of concentric vs. eccentric actions after consistent, regimented RT. Meta-regression was used to quantify and compare the magnitude of effects between conditions, as well as to determine the potential influence of covariates on findings.  (Introduction para. 4) |
| Williams et al. (2017)  [22] | The primary aim of this study was to examine the previous literature comparing periodized resistance training plans to non-periodized resistance training plans and determine a quantitative estimate of effect on maximal strength.  (Abstract) |
| Buskard et al. (2018)  [23] | The purpose of this study was to determine whether SME [supramaximal eccentric training] elicits greater lower-body strength improvements than TRAD [traditional methods].  (Abstract) |
| Domingos and Polito (2018)  [24] | The aim of this study was to compare, by means of a systematic review and meta-analysis, the effects of resistance training with and without blood flow restriction (BFR) on blood pressure (BP).  (Abstract) |
| Grgic, Lazinica, et al. (2018)  [25] | Our goal was to systematically review the current literature and interpret the findings regarding the effects of periodized (PER) versus non-periodized (NP) resistance training programs aimed at muscular hypertrophy.  (Abstract) |
| Grgic, Schoenfeld, Davies, et al. (2018)  [26] | [T]he purpose of the present paper is threefold: (1) to perform a systematic review of the studies that compare different RT frequencies while assessing muscular strength outcomes; (2) to quantify the findings with a meta-analysis; and (3) to draw evidence-based conclusions guiding exercise program design.  (Introduction para. 2) |
| Grgic, Schoenfeld, Skrepnik, et al. (2018)  [27] | [T]he intention of this review is twofold: (i) to aggregate findings and interpret the studies that assessed muscular strength adaptations to different RI [rest interval] durations, and (ii) to provide evidence-based recommendations for exercise practitioners and athletes. Such a treatise will be useful to coaches, athletes, and to a wider community participating in resistance-training activities with a goal of increasing muscular strength.  (Introduction para. 5) |
| Hackett et al. (2018)  [28] | The purpose of this systematic review of literature was to investigate the effect of movement velocity during resistance training on muscle hypertrophy.  (Abstract) |
| Lixandrão et al. (2018)  [29] | To compare the effects of HL (high intensity)- versus BFR-RT (blood-flow restriction resistance training) on muscle adaptations using a systematic review and meta-analysis procedure.  (Abstract) |
| Ralston et al. (2018)  [30] | We conducted a meta-analysis that (1) quantified the effects of low (LF; 1 day week^− 1^), medium (MF; 2 days week^− 1^), or high (HF; ≥ 3 days week^− 1^) RT frequency on muscular strength per exercise; (2) examined the effects of different RT frequency on one repetition maximum (1RM) strength gain profiles (multi-joint exercises and single joint exercises); (3) examined the effects of different RT frequency on 1RM strength gain when RT volume is equated; and (4) examined the effects of different RT frequency on 1RM strength gains on upper and lower body.  (Abstract) |
| Ramos-Campo et al. (2018)  [31] | [T]he aim of this work was to systematically review the studies which have investigated using RTH [resistance training in hypoxia] to improve muscular size and strength, and to perform a meta-analysis to determine the effect of RTH on these adaptive parameters.  (Introduction para. 3) |
| da Rosa Orssatto et al. (2019)  [32] | [T]he aim of this systematic review and meta-analysis was to compare the magnitude of functional capacity improvements following fast-intended velocity vs. moderate velocity resistance training for lower-limbs in older adults.  (Introduction para. 4) |
| Vicens-Bordas et al., 2018  [33] | The primary aim of this systematic review was to determine if inertial flywheel resistance training is superior to gravity-dependent resistance training in improving muscle strength.  (Introduction para. 5) |
| Grgic et al. (2019)  [34] | Given the overall lack of consensus on the effects of temporal specificity of resistance training, the present paper endeavored to elucidate this topic by conducting a systematic review and a meta-analysis of studies that examined time of day-specific resistance training and its effects on muscle strength and hypertrophy.  (Introduction para. 5) |
| Hansen et al. (2019)  [35] | The aim of this systematic review is therefore to discuss this line of evidence, which is in contrast to current clinical practice, and to re-open the debate as to what dynamic strength training intensities should actually be applied.  (Abstract) |
| Krzysztofik et al. (2019)  [36] | The purpose of the present paper was to provide an objective and critical review related to advanced RT [resistance training] methods and techniques influencing skeletal muscle, which may contribute to maximizing muscle hypertrophy in both recreational and competitive athletes.  (Introduction para. 3) |
| Latella, Grgic, et al. (2019)  [37] | [T]he the present article aims to review and collate the available evidence on interset interventions and evaluate their effect on acute resistance training performance and associated physiological responses. As a secondary aim, we have endeavored to provide evidence-based recommendations for exercise professionals to optimize the interset period based on the collective findings of this review.  (Introduction para. 2) |
| Latella, Teo, et al. (2019)  [38] | The objective of this investigation was to determine the efficacy of a single session of CSs [cluster sets] to attenuate losses in force, velocity and power compared to traditional set (TS) training.  (Abstract) |
| Lopes et al. (2019)  [39] | [T]he objective of this study was to describe the effects of ERT [elastic resistance training] training compared to CRT training on muscular strength, in different population profiles.  (Introduction para. 5) |
| Molinari et al. (2019)  [40] | [T]he aim of this systematic review and meta-analysis was to compare eccentric-focused training (ET) and CT [conventional training] on the muscular strength of older adults’ lower limbs.  (Introduction para. 5) |
| Ralston et al. (2019)  [41] | The purpose of this review and meta-analysis, therefore, was four-fold: (1) to reexamine the effects of RT volume (S or M3) of ST on muscular strength per exercise; (2) to determine if specific set-volume (S [single set] vs. M3 [three-set]) produce different strength gains when multi-joint exercises are compared with single-joint exercises; (3) to investigate if the magnitude of strength gain differs between multi-joint and single-joint exercises by population group (trained vs. untrained) and body segmentations (upper vs. lower body). The final objective; (4) is to provide a perspective on developing muscular strength that provides recommendations on daily RT set-volume. for pre-flight strength development.  (Introduction para. 8) |
| Schoenfeld et al. (2019)  [42] | The purpose of this paper was to conduct a systematic review and meta-analysis of experimental studies designed to investigate the effects of weekly training frequency on hypertrophic adaptations.  (Abstract) |
| Androulakis-Korakakis et al. (2020)  [43] | [T]he present article aims to systematically review the available evidence regarding the minimum effective training dose required to increase 1RM strength in resistance-trained men.  (Introduction para. 5) |
| Centner and Lauber (2020)  [44] | To summarize the existing evidence on the long-term effects of low-load (LL) blood flow restricted (BFR) exercise on neural markers including both central and peripheral adaptations.  (Abstract) |
| Cuyul-Vásquez et al. (2020)  [45] | The aim of this systematic review with meta-analysis was to compare the effectiveness of resistance exercise alone versus resistance exercise with BFR for pain relief and functional improvement in patients with knee pain.  (Introduction para. 4) |
| Ferlito et al. (2020)  [46] | (1) What are the BFR [blood flow restriction] effects in individuals with knee osteoarthritis compared to high or low load training?  (2) BFR’s different forms of application influence this population’s physical therapy rehabilitation results?  (Introduction para. 5) |
| Grgic (2020)  [47] | [T]he present paper aimed to perform a meta-analysis on the effects of low-load vs. high-load resistance training on type I and type II muscle fiber hypertrophy.  (Introduction para. 4) |
| Grønfeldt et al. (2020)  [48] | [T]he aim of the present analysis was to perform a systematic review and meta-analysis to compare the effect of low-load blood-flow-restricted training versus conventional high-load strength training on maximal muscle strength in healthy individuals.  (Introduction para. 5) |
| João et al. (2020)  [49] | Can intensity in strength training change caloric expenditure? (Title)  This review aimed to investigate the effect of ST [strength training] on EE (energy expenditure) in adults using a systematic literature review and subsequent meta-analysis.  (Abstract) |
| Jukic et al. (2020)  [50] | This study aimed to systematically review and meta-analyse current evidence on the differences between traditional and alternative (cluster and rest redistribution) set structures on acute mechanical, metabolic, and perceptual responses during and after RT, and to discuss potential reasons for the disparities noted in the literature.  (Abstract) |
| Schoenfeld and Grgic (2020)  [51] | [T]he purpose of this article was to systematically review the literature as to the effects of performing exercise with a full versus partial ROM [range of motion] during dynamic, longitudinal RT [resistance training] programs on changes in muscle hypertrophy.  (Introduction para. 5) |
| Souza et al. (2020)  [52] | To compare the effects of high-load (≥ 70 of 1RM) and low-load (< 70 of 1RM) resistance training (RT) on femoral neck and lumbar spine bone mineral density (BMD) in middle-aged and older people.  (Abstract) |
| Baz-Valle et al. (2021)  [53] | [T]he aim of this systematic review was to analyze training programs with different arrangement of number of sets, repetition range, and training frequency to determine whether assessing the total number of sets is a valid method to quantify training volume in the context of hypertrophy training.  (Introduction para. 5) |
| Cerqueira, Lira, et al. (2021)  [54] | The purpose of this study was to use the systematic review and meta-analytical approach to investigate the effects of LL [low load]-BFR [blood flow restriction] compared with LL-RT [resistance training] on the number of repetitions to achieve repetition failure.  (Introduction para. 3) |
| Cerqueira, Maciel, et al. (2021)  [55] | To compare the short- and long-term effects of low-load resistance training with blood-flow restriction (LL-BFR) versus low- (LL-RT) or high- (HL-RT) load resistance training with free blood flow on myoelectric activity and investigate the differences between failure (exercise performed to volitional failure) and nonfailure (exercise not performed to volitional failure) protocols.  (Abstract) |
| Cuthbert et al. (2021)  [56] | The aim of this review was to investigate the effect of training frequency on muscular strength to determine if a potential method to accommodate in-season resistance training, during busy training schedules, could be achieved by utilizing shorter more frequent training sessions across a training week.  (Abstract) |
| da Silva et al. (2021)  [57] | [T]he present study aims to conduct a literature review aimed at identifying, and analyzing the objectives, and evaluation methods for the responses after different recovery intervals in resistance training.  (Introduction para. 7) |
| Davies et al. (2021)  [58] | The aim of this review was to collate evidence from the available literature that directly compared the effects of RT programs implementing cluster and traditional set configurations on human muscular and neuromuscular adaptations.  (Section 1.2) |
| de Queiros et al. (2021)  [59] | The aim of this study was to systematically review and pool the available evidence on the differences in neuromuscular and metabolic responses at LL-RE [low load-resistance exercise] with different pressure of BFR [blood flow restriction].  (Abstract) |
| Grgic et al. (2021)  [60] | [T]his review aimed to perform a meta-analysis examining: (a) acute effects of adopting an internal focus vs. external focus of attention on muscular strength; and (b) long-term effects of adopting an internal focus vs. external focus of attention during resistance training on gains in muscular strength. Such an analysis would be of practical relevance given the importance of muscular strength for athletic performance and activities of daily living.  (Introduction para. 3) |
| Grgic and Mikulic (2021)  [61] | [T]his review aimed to perform a meta-analysis examining the effects of external focus vs. internal focus vs. control on muscular endurance.  (Introduction para. 4) |
| Heidel et al. (2021)  [62] | [T]he purpose of this systematic review and meta-analysis was to examine original training studies that compared the efficacy of a free-weight resistance training intervention with that of a machine-based resistance training intervention.  (p. 1062) |
| Jukic et al. (2021)  [63] | The aim of this systematic review and meta-analysis was to synthesise the available evidence on the effectiveness of traditional (TS), cluster (CS) and rest redistribution (RR) set structures in promoting chronic RT adaptations, and provide an overview of the factors which might differentially influence the magnitude of specific training adaptations between set structure types.  (Abstract) |
| Lacio et al. (2021)  [64] | The aim of this study was to systematically review the literature and compare the effects of resistance training that was performed with low loads versus moderate and high loads in untrained and trained healthy adult males on the development of maximal strength and muscle hypertrophy during randomized experimental designs.  (Abstract) |
| Larsen et al. (2021)  [65] | This review aimed to summarise the effects of different subjective and objective autoregulation methods for intensity and volume on enhancing maximal strength.  (Abstract) |
| Liao et al. (2021)  [66] | The purpose of this study was to determine whether VBT [velocity-based training] was more effective than PBT [1RM percentage-based training] in enhancing strength, jump, linear sprint and CODs [change of direction speed].  (Introduction para. 4) |
| Liu et al. (2021)  [67] | In this meta-analysis, the aims were to examine the effects of resistance training with and without blood flow restriction on vascular function.  (Abstract) |
| Lopez et al. (2021)  [68] | This study aimed to analyze the effect of resistance training (RT) performed until volitional failure with low, moderate, and high loads on muscle hypertrophy and muscle strength in healthy adults and to assess the possible participant-, design-, and training-related covariates that may affect the adaptations.  (Abstract) |
| Marshall et al. (2021)  [69] | The aim of this systematic review and meta-analysis was to compare the acute and chronic responses of lower body cluster, contrast, complex, and traditional training across a range of athletic performance outcomes (1-repetition maximum squat strength, jump height, peak power, peak force, peak velocity, and sprint time).  (Abstract) |
| Moran et al. (2021)  [70] | [T]he aim of this systematic review and meta-analysis was to evaluate the effects of BLE and ULE on movement speed such as short sprinting or change of direction-type movements. Our objective was to help to determine the relative effectiveness of these training types to better inform coaches’ programming choices with regard to clarifying the specificity of the training stimulus.  (Introduction para. 4) |
| Nitzsche et al. (2021)  [71] | This meta-analysis aimed to evaluate the effectiveness of low-load Resistance Training (RT) with or without Blood Flow Restriction (BFR) compared with conventional RT on muscle strength in open and closed kinetic chains, muscle volume and pain in individuals with orthopaedic impairments.  (Abstract) |
| Nunes et al. (2021)  [72] | The objectives of this paper were to: (a) systematically review studies that explored the effects of exercise order (EO) on muscular strength and/or hypertrophy; (b) pool their results using a meta-analysis; and (c) provide recommendations for the prescription of EO in resistance training (RT) programmes.  (Abstract) |
| Pallarés et al. (2021)  [73] | To conduct a systematic review and meta-analysis of the scientific evidence examining the effects of full and partial ROM [range of motion] resistance training interventions on neuromuscular, functional, and structural adaptations.  (Abstract) |
| Refalo et al. (2021)  [74] | This systematic review and meta-analysis therefore aimed to further elucidate the role of RT load in developing various indices of maximal strength (i.e., dynamic 1-RM, isometric, and isokinetic strength), muscle hypertrophy (i.e., lean body/fat-free mass, and both whole-muscle and muscle fibre CSA), and sport-specific or neuromuscular task performance.  (Introduction para. 6) |
| Ribeiro et al. (2021)  [75] | [T]he purpose of this systematic review was to synthesize and analyse research findings on the effects of warm-up strategies on strength performance during resistance exercises.  (Introduction para. 3) |
| Rodrigo-Mallorca et al. (2021)  [76] | [T]he present systematic review and meta-analysis aimed to determine the effectiveness of the low-intensity resistance training with blood flow restriction compared to dynamic high-intensity resistance training on strength and muscle mass in non-active older adults.  (Introduction para. 4) |
| Vieira et al. (2021)  [77] | [T]he purpose of this study was to systematically review randomized and nonrandomized longitudinal studies on the effects of RT [resistance training] performed to concentric failure or not to failure on muscle strength, hypertrophy, and maximal power output in healthy young subjects and in older adults.  (Introduction para. 5) |
| Zhang et al. (2021)  [78] | (1) examine the difference between the two training methods;  (2) reveal their functions in different training events and interventions;  (3) quantify the differences among the APRE [Autoregulatory Progressive Resistance Exercise], RPE [Rating of Perceived Exertion], and VBT [Velocity-Based Training] programs in maximum strength training by synthesizing evidence from current published studies.  (Introduction para. 8) |
| Baz-Valle et al. (2022)  [79] | The main goal of this study was to compare responses to moderate and high training volumes aimed at inducing muscle hypertrophy.  (Abstract) |
| Grgic et al. (2022)  [80] | [I]n this review, we performed an updated meta-analysis exploring the effects of training to failure on muscular  strength as well as conducted the first meta-analysis exploring the effects of training to muscle failure on hypertrophy outcomes.  (Introduction para. 3) |
| Carvalho et al. (2022)  [81] | The purpose of this paper was to conduct a systematic review and meta-analysis of studies that compared muscle hypertrophy and strength gains between resistance training protocols employing very low (VLL < 30% of 1-repetition maximum (RM) or >35RM), low (LL30%-59% of 1RM, or 16-35RM), moderate (ML60%-79% of 1RM, or 8-15RM), and high (HL ≥ 80% of 1RM, or ≤ 7RM) loads with matched volume loads (sets 🞪 repetitions 🞪 weight).  (Abstract) |
| Hackett et al. (2022)  [82] | To examine the effect of total repetitions per set on local muscular endurance (LME) assessed via maximal repetitions to concentric muscular failure using loads based on a percentage of pre-intervention one-repetition maximum (%1RM_PRE_) and post-intervention 1RM (%1RM_POST_).  (Abstract) |
| Hickmott et al. (2022)  [83] | The primary purpose of this review was to determine the chronic effects of load and volume autoregulation on 1RM strength adaptations, with cross-sectional area (CSA) muscle hypertrophy as a secondary outcome.  (“Objectives”) |
| Kassiano et al. (2022)  [84] | [T]his review aimed to systematically review the effects of exercise variation on muscle hypertrophy and strength to draw practical conclusions for prescription and provide suggestions for future research.  (Introduction para. 3) |
| Koc et al. (2022)  [85] | The present systematic review aimed to investigate the effect of LL-BFR [low load-blood flow restriction] training on quadriceps strength, quadriceps mass, knee joint pain, and ACL [anterior cruciate ligament] graft laxity after ACL reconstruction compared to non-BFR training.  (Abstract) |
| Moesgaard et al. (2022)  [86] | The aims of this study were to examine the current body of literature to determine whether there is an effect of periodization of training volume and intensity on maximal strength and muscle hypertrophy, and, if so, to determine how these variables are more effectively periodized to promote increases in strength and muscle hypertrophy, when volume is equated between conditions from pre to post intervention.  (Abstract) |
| Orange et al. (2022)  [87] | [W]e systematically reviewed, meta-analysed, and appraised the quality of evidence regarding the effects of VBT vs. traditional resistance training methods on adaptations in strength, power, and linear sprint speed.  (Introduction para. 6) |
| Vieira et al. (2022)  [88] | [T]he aim of this systematic review and meta-analysis was to compare the effects of RTF [resistance training to failure] versus RTNF [resistance training not to failure] on acute fatigue.  (Abstract) |
| Zhang et al. (2022)  [89] | The purpose was to analyze the comparison of velocity-based resistance training and one-repetition maximum (%1RM) percentage-based training in maximal strength improvement by meta-analyzing and to find the reasons for the controversial findings of different studies.  (Abstract) |

## References

1. Durall CJ, Hermsen D, Demuth C. Systematic review of single-set versus multiple-set resistance-training randomized controlled trials: Implications for rehabilitation. Crit Rev Phys Rehabil Med. 2006;18(2):107-16.

2. Bågenhammar S, Hansson EE. Repeated sets or single set of resistance training - A systematic review. Adv Physiother. 2007;9(4):154-60.

3. Krieger JW. Single versus multiple sets of resistance exercise: A Meta-regression. J Strength Cond Res. 2009;23(6):1890-901.

4. Roig M, O'Brien K, Kirk G, Murray R, McKinnon P, Shadgan B, et al. The effects of eccentric versus concentric resistance training on muscle strength and mass in healthy adults: A systematic review with meta-analysis. Br J Sports Med. 2009;43(8):556-68.

5. Krieger JW. Single vs. multiple sets of resistance exercise for muscle hypertrophy: A meta-analysis. J Strength Cond Res. 2010;24(4):1150-9.

6. Tschopp M, Sattelmayer MK, Hilfiker R. Is power training or conventional resistance training better for function in elderly persons? A meta-analysis. Age Ageing. 2011;40(5):549-56.

7. Raymond MJ, Bramley-Tzerefos RE, Jeffs KJ, Winter A, Holland AE. Systematic review of high-intensity progressive resistance strength training of the lower limb compared with other intensities of strength training in older adults. Arch Phys Med Rehabil. 2013;94(8):1458-72.

8. Harries SK, Lubans DR, Callister R. Systematic review and meta-analysis of linear and undulating periodized resistance training programs on muscular strength. J Strength Cond Res. 2015;29(4):1113-25.

9. Schoenfeld BJ, Ogborn DI, Krieger JW. Effect of Repetition Duration During Resistance Training on Muscle Hypertrophy: A Systematic Review and Meta-Analysis. Sports Med. 2015;45(4):577-85.

10. Csapo R, Alegre LM. Effects of resistance training with moderate vs heavy loads on muscle mass and strength in the elderly: A meta-analysis. Scand J Med Sci Sports. 2016;26(9):995-1006.

11. Davies T, Orr R, Halaki M, Hackett D. Effect of Training Leading to Repetition Failure on Muscular Strength: A Systematic Review and Meta-Analysis. Sports Med. 2016;46(4):487-502.

12. Schoenfeld BJ, Ogborn D, Krieger JW. Effects of Resistance Training Frequency on Measures of Muscle Hypertrophy: A Systematic Review and Meta-Analysis. Sports Med. 2016;46(11):1689-97.

13. Schoenfeld BJ, Wilson JM, Lowery RP, Krieger JW. Muscular adaptations in low- versus high-load resistance training: A meta-analysis. Eur J Sport Sci. 2016;16(1):1-10.

14. Davies TB, Kuang K, Orr R, Halaki M, Hackett D. Effect of Movement Velocity During Resistance Training on Dynamic Muscular Strength: A Systematic Review and Meta-Analysis. Sports Med. 2017;47(8):1603-17.

15. Douglas J, Pearson S, Ross A, McGuigan M. Chronic Adaptations to Eccentric Training: A Systematic Review. Sports Med. 2017;47(5):917-41.

16. Grgic J, Lazinica B, Mikulic P, Krieger JW, Schoenfeld BJ. The effects of short versus long inter-set rest intervals in resistance training on measures of muscle hypertrophy: A systematic review. Eur J Sport Sci. 2017;17(8):983-93.

17. Grgic J, Mikulic P, Podnar H, Pedisic Z. Effects of linear and daily undulating periodized resistance training programs on measures of muscle hypertrophy: A systematic review and meta-analysis. PeerJ. 2017;2017(8).

18. Ralston GW, Kilgore L, Wyatt FB, Baker JS. The Effect of Weekly Set Volume on Strength Gain: A Meta-Analysis. Sports Med. 2017;47(12):2585-601.

19. Schoenfeld BJ, Grgic J, Ogborn D, Krieger JW. Strength and hypertrophy adaptations between low- vs. High-load resistance training: A systematic review and meta-analysis. J Strength Cond Res. 2017;31(12):3508-23.

20. Schoenfeld BJ, Ogborn D, Krieger JW. Dose-response relationship between weekly resistance training volume and increases in muscle mass: A systematic review and meta-analysis. J Sports Sci. 2017;35(11):1073-82.

21. Schoenfeld BJ, Ogborn DI, Vigotsky AD, Franchi MV, Krieger JW. Hypertrophic Effects of Concentric vs. Eccentric Muscle Actions: A Systematic Review and Meta-analysis. J Strength Cond Res. 2017;31(9):2599-608.

22. Williams TD, Tolusso DV, Fedewa MV, Esco MR. Comparison of Periodized and Non-Periodized Resistance Training on Maximal Strength: A Meta-Analysis. Sports Med. 2017;47(10):2083-100.

23. Buskard ANL, Gregg HR, Ahn S. Supramaximal Eccentrics Versus Traditional Loading in Improving Lower-Body 1RM: A Meta-Analysis. Res Q Exerc Sport. 2018;89(3):340-6.

24. Domingos E, Polito MD. Blood pressure response between resistance exercise with and without blood flow restriction: A systematic review and meta-analysis. Life Sci. 2018;209:122-31.

25. Grgic J, Lazinica B, Mikulic P, Schoenfeld BJ. Should resistance training programs aimed at muscular hypertrophy be periodized? A systematic review of periodized versus non-periodized approaches. Sci Sports. 2018;33(3):e97-e104.

26. Grgic J, Schoenfeld BJ, Davies TB, Lazinica B, Krieger JW, Pedisic Z. Effect of Resistance Training Frequency on Gains in Muscular Strength: A Systematic Review and Meta-Analysis. Sports Med. 2018;48(5):1207-20.

27. Grgic J, Schoenfeld BJ, Skrepnik M, Davies TB, Mikulic P. Effects of Rest Interval Duration in Resistance Training on Measures of Muscular Strength: A Systematic Review. Sports Med. 2018;48(1):137-51.

28. Hackett DA, Davies TB, Orr R, Kuang K, Halaki M. Effect of movement velocity during resistance training on muscle-specific hypertrophy: A systematic review. Eur J Sport Sci. 2018;18(4):473-82.

29. Lixandrão ME, Ugrinowitsch C, Berton R, Vechin FC, Conceição MS, Damas F, et al. Magnitude of Muscle Strength and Mass Adaptations Between High-Load Resistance Training Versus Low-Load Resistance Training Associated with Blood-Flow Restriction: A Systematic Review and Meta-Analysis. Sports Med. 2018;48(2):361-78.

30. Ralston GW, Kilgore L, Wyatt FB, Buchan D, Baker JS. Weekly Training Frequency Effects on Strength Gain: A Meta-Analysis. Sports Med - Open. 2018;4(1).

31. Ramos-Campo DJ, Scott BR, Alcaraz PE, Rubio-Arias JA. The efficacy of resistance training in hypoxia to enhance strength and muscle growth: A systematic review and meta-analysis. Eur J Sport Sci. 2018;18(1):92-103.

32. da Rosa Orssatto LB, de la Rocha Freitas C, Shield AJ, Silveira Pinto R, Trajano GS. Effects of resistance training concentric velocity on older adults' functional capacity: A systematic review and meta-analysis of randomised trials. Exp Gerontol. 2019;127.

33. Vicens-Bordas J, Esteve E, Fort-Vanmeerhaeghe A, Bandholm T, Thorborg K. Is inertial flywheel resistance training superior to gravity-dependent resistance training in improving muscle strength? A systematic review with meta-analyses. J Sci Med Sport. 2018;21(1):75-83.

34. Grgic J, Lazinica B, Garofolini A, Schoenfeld BJ, Saner NJ, Mikulic P. The effects of time of day-specific resistance training on adaptations in skeletal muscle hypertrophy and muscle strength: A systematic review and meta-analysis. Chronobiol Int. 2019;36(4):449-60.

35. Hansen D, Abreu A, Doherty P, Völler H. Dynamic strength training intensity in cardiovascular rehabilitation: is it time to reconsider clinical practice? A systematic review. Eur J Prev Cardiol. 2019;26(14):1483-92.

36. Krzysztofik M, Wilk M, Wojdała G, Gołaś A. Maximizing muscle hypertrophy: A systematic review of advanced resistance training techniques and methods. Int J Environ Res Public Health. 2019;16(24).

37. Latella C, Grgic J, Der Westhuizen DV. Effect of interset strategies on acute resistance training performance and physiological responses: A systematic review. J Strength Cond Res. 2019;33:S180-S93.

38. Latella C, Teo WP, Drinkwater EJ, Kendall K, Haff GG. The Acute Neuromuscular Responses to Cluster Set Resistance Training: A Systematic Review and Meta-Analysis. Sports Med. 2019;49(12):1861-77.

39. Lopes JSS, Machado AF, Micheletti JK, de Almeida AC, Cavina AP, Pastre CM. Effects of training with elastic resistance versus conventional resistance on muscular strength: A systematic review and meta-analysis. SAGE Open Med. 2019;7:2050312119831116.

40. Molinari T, Steffens T, Roncada C, Rodrigues R, Dias CP. Effects of eccentric-focused versus conventional training on lower limb muscular strength in older adults: A systematic review with meta-analysis. J Aging Phys Act. 2019;27(6):823-30.

41. Ralston GW, Kilgore L, Wyatt FB, Dutheil F, Jaekel P, Buchan DS, et al. Re-examination of 1- vs. 3-Sets of Resistance Exercise for Pre-spaceflight Muscle Conditioning: A Systematic Review and Meta-Analysis. Front Physiol. 2019;10:864.

42. Schoenfeld BJ, Grgic J, Krieger J. How many times per week should a muscle be trained to maximize muscle hypertrophy? A systematic review and meta-analysis of studies examining the effects of resistance training frequency. J Sports Sci. 2019;37(11):1286-95.

43. Androulakis-Korakakis P, Fisher JP, Steele J. The Minimum Effective Training Dose Required to Increase 1RM Strength in Resistance-Trained Men: A Systematic Review and Meta-Analysis. Sports Med. 2020;50(4):751-65.

44. Centner C, Lauber B. A Systematic Review and Meta-Analysis on Neural Adaptations Following Blood Flow Restriction Training: What We Know and What We Don't Know. Front Physiol. 2020;11:887.

45. Cuyul-Vásquez I, Leiva-Sepúlveda A, Catalán-Medalla O, Araya-Quintanilla F, Gutiérrez-Espinoza H. The addition of blood flow restriction to resistance exercise in individuals with knee pain: a systematic review and meta-analysis. Braz J Phys Ther. 2020.

46. Ferlito JV, Pecce SAP, Oselame L, De Marchi T. The blood flow restriction training effect in knee osteoarthritis people: a systematic review and meta-analysis. Clinical Rehabilitation. 2020;34(11):1378-90.

47. Grgic J. The Effects of Low-Load vs. High-Load Resistance Training on Muscle Fiber Hypertrophy: A Meta-Analysis. J Hum Kinet. 2020;74(1):51-8.

48. Grønfeldt BM, Lindberg Nielsen J, Mieritz RM, Lund H, Aagaard P. Effect of blood-flow restricted vs heavy-load strength training on muscle strength: Systematic review and meta-analysis. Scand J Med Sci Sports. 2020;30(5):837-48.

49. João GA, Rodriguez D, Tavares LD, Carvas Junior N, Miranda ML, Reis VM, et al. Can intensity in strength training change caloric expenditure? Systematic review and meta-analysis. Clin Physiol Funct Imaging. 2020;40(2):55-66.

50. Jukic I, Ramos AG, Helms ER, McGuigan MR, Tufano JJ. Acute Effects of Cluster and Rest Redistribution Set Structures on Mechanical, Metabolic, and Perceptual Fatigue During and After Resistance Training: A Systematic Review and Meta-analysis. Sports Med. 2020;50(12):2209-36.

51. Schoenfeld BJ, Grgic J. Effects of range of motion on muscle development during resistance training interventions: A systematic review. SAGE Open Med. 2020;8:2050312120901559.

52. Souza D, Barbalho M, Ramirez-Campillo R, Martins W, Gentil P. High and low-load resistance training produce similar effects on bone mineral density of middle-aged and older people: A systematic review with meta-analysis of randomized clinical trials. Exp Gerontol. 2020;138.

53. Baz-Valle E, Fontes-Villalba M, Santos-Concejero J. Total Number of Sets as a Training Volume Quantification Method for Muscle Hypertrophy: A Systematic Review. J Strength Cond Res. 2021;35(3):870-8.

54. Cerqueira MS, Lira M, Mendonça Barboza JA, Burr JF, Wanderley ELTB, Maciel DG, et al. Repetition Failure Occurs Earlier During Low-Load Resistance Exercise With High But Not Low Blood Flow Restriction Pressures: A Systematic Review and Meta-analysis. J Strength Cond Res. 2021.

55. Cerqueira MS, Maciel DG, Barboza JAM, Centner C, Lira M, Pereira R, et al. Effects of low-load blood flow restriction exercise to failure and non-failure on myoelectric activity: a meta-analysis. J Athl Train. 2021;57(4):402-17.

56. Cuthbert M, Haff GG, Arent SM, Ripley N, McMahon JJ, Evans M, et al. Effects of Variations in Resistance Training Frequency on Strength Development in Well-Trained Populations and Implications for In-Season Athlete Training: A Systematic Review and Meta-analysis. Sports Med. 2021;51(9):1967-82.

57. da Silva JB, de Lima e Silva L, Nunes RAM, Lopes GC, de Mello DB, Lima VP, et al. Evaluation methods and objectives for neuromuscular and hemodynamic responses subsequent to different rest intervals in resistance training: A systematic review. Arch Med Deporte. 2021;38(3):180-4.

58. Davies TB, Tran DL, Hogan CM, Haff GG, Latella C. Chronic Effects of Altering Resistance Training Set Configurations Using Cluster Sets: A Systematic Review and Meta-Analysis. Sports Med. 2021;51(4):707-36.

59. de Queiros VS, de França IM, Trybulski R, Vieira JG, dos Santos IK, Neto GR, et al. Myoelectric Activity and Fatigue in Low-Load Resistance Exercise With Different Pressure of Blood Flow Restriction: A Systematic Review and Meta-Analysis. Front Physiol. 2021;12.

60. Grgic J, Mikulic I, Mikulic P. Acute and long-term effects of attentional focus strategies on muscular strength: A meta-analysis. Sports. 2021;9(11).

61. Grgic J, Mikulic P. Effects of Attentional Focus on Muscular Endurance: A Meta-Analysis. Int J Environ Res Public Health. 2021;19(1).

62. Heidel KA, Novak ZJ, Dankel SJ. Machines and free weight exercises: a systematic review and meta-analysis comparing changes in muscle size, strength, and power. J Sports Med Phys Fitness. 2021.

63. Jukic I, Van Hooren B, Ramos AG, Helms ER, McGuigan MR, Tufano JJ. The Effects of Set Structure Manipulation on Chronic Adaptations to Resistance Training: A Systematic Review and Meta-Analysis. Sports Med. 2021;51(5):1061-86.

64. Lacio M, Vieira JG, Trybulski R, Campos Y, Santana D, Filho JE, et al. Effects of resistance training performed with different loads in untrained and trained male adult individuals on maximal strength and muscle hypertrophy: A systematic review. Int J Environ Res Public Health. 2021;18(21).

65. Larsen S, Kristiansen E, van den Tillaar R. Effects of subjective and objective autoregulation methods for intensity and volume on enhancing maximal strength during resistance-training interventions: A systematic review. PeerJ. 2021;9.

66. Liao KF, Wang XX, Han MY, Li LL, Nassis GP, Li YM. Effects of velocity based training vs. traditional 1RM percentage-based training on improving strength, jump, linear sprint and change of direction speed performance: A Systematic review with meta-analysis. PLoS ONE. 2021;16(11 November).

67. Liu Y, Jiang N, Pang F, Chen T. Resistance Training with Blood Flow Restriction on Vascular Function: A Meta-analysis. Int J Sports Med. 2021;42(7):577-87.

68. Lopez P, Radaelli R, Taaffe DR, Newton RU, Galvão DA, Trajano GS, et al. Resistance Training Load Effects on Muscle Hypertrophy and Strength Gain: Systematic Review and Network Meta-analysis. Medicine and Science in Sports and Exercise. 2021;53(6):1206-16.

69. Marshall J, Bishop C, Turner A, Haff GG. Optimal Training Sequences to Develop Lower Body Force, Velocity, Power, and Jump Height: A Systematic Review with Meta-Analysis. Sports Med. 2021;51(6):1245-71.

70. Moran J, Ramirez-Campillo R, Liew B, Chaabene H, Behm DG, García-Hermoso A, et al. Effects of Bilateral and Unilateral Resistance Training on Horizontally Orientated Movement Performance: A Systematic Review and Meta-analysis. Sports Med. 2021;51(2):225-42.

71. Nitzsche N, Stäuber A, Tiede S, Schulz H. The effectiveness of blood-flow restricted resistance training in the musculoskeletal rehabilitation of patients with lower limb disorders: A systematic review and meta-analysis. Clinical Rehabilitation. 2021;35(9):1221-34.

72. Nunes JP, Grgic J, Cunha PM, Ribeiro AS, Schoenfeld BJ, de Salles BF, et al. What influence does resistance exercise order have on muscular strength gains and muscle hypertrophy? A systematic review and meta-analysis. Eur J Sport Sci. 2021;21(2):149-57.

73. Pallarés JG, Hernández-Belmonte A, Martínez-Cava A, Vetrovsky T, Steffl M, Courel-Ibáñez J. Effects of range of motion on resistance training adaptations: A systematic review and meta-analysis. Scand J Med Sci Sports. 2021;31(10):1866-81.

74. Refalo MC, Hamilton DL, Paval DR, Gallagher IJ, Feros SA, Fyfe JJ. Influence of resistance training load on measures of skeletal muscle hypertrophy and improvements in maximal strength and neuromuscular task performance: A systematic review and meta-analysis. J Sports Sci. 2021;39(15):1723-45.

75. Ribeiro B, Pereira A, Neves P, Marinho D, Marques M, Neiva HP. The effect of warm-up in resistance training and strength performance: A systematic review. Motricidade. 2021;17:87-94.

76. Rodrigo-Mallorca D, Loaiza-Betancur AF, Monteagudo P, Blasco-Lafarga C, Chulvi-Medrano I. Resistance training with blood flow restriction compared to traditional resistance training on strength and muscle mass in non-active older adults: A systematic review and meta-analysis. Int J Environ Res Public Health. 2021;18(21).

77. Vieira AF, Umpierre D, Teodoro JL, Lisboa SC, Baroni BM, Izquierdo M, et al. Effects of Resistance Training Performed to Failure or Not to Failure on Muscle Strength, Hypertrophy, and Power Output: A Systematic Review With Meta-Analysis. J Strength Cond Res. 2021;35(4):1165-75.

78. Zhang X, Li H, Bi S, Luo Y, Cao Y, Zhang G. Auto-Regulation Method vs. Fixed-Loading Method in Maximum Strength Training for Athletes: A Systematic Review and Meta-Analysis. Front Physiol. 2021;12.

79. Baz-Valle E, Balsalobre-Fernández C, Alix-Fages C, Santos-Concejero J. A Systematic Review of the Effects of Different Resistance Training Volumes on Muscle Hypertrophy. J Hum Kinet. 2022;81(1):199-210.

80. Grgic J, Schoenfeld BJ, Orazem J, Sabol F. Effects of resistance training performed to repetition failure or non-failure on muscular strength and hypertrophy: A systematic review and meta-analysis. J Sport Health Sci. 2022;11(2):202-11.

81. Carvalho L, Junior RM, Barreira J, Schoenfeld BJ, Orazem J, Barroso R. Muscle hypertrophy and strength gains after resistance training with different volume-matched loads: a systematic review and meta-analysis. Appl Physiol Nutr Metab. 2022;47(4):357-68.

82. Hackett DA, Ghayomzadeh M, Farrell SN, Davies TB, Sabag A. Influence of total repetitions per set on local muscular endurance: A systematic review with meta-analysis and meta-regression. Sci Sports. 2022.

83. Hickmott LM, Chilibeck PD, Shaw KA, Butcher SJ. The Effect of Load and Volume Autoregulation on Muscular Strength and Hypertrophy: A Systematic Review and Meta-Analysis. Sports Med - Open. 2022;8(1).

84. Kassiano W, Nunes JP, Costa B, Ribeiro AS, Schoenfeld BJ, Cyrino ES. Does Varying Resistance Exercises Promote Superior Muscle Hypertrophy and Strength Gains? A Systematic Review. J Strength Cond Res. 2022.

85. Koc BB, Truyens A, Heymans MJLF, Jansen EJP, Schotanus MGM. Effect of Low-Load Blood Flow Restriction Training After Anterior Cruciate Ligament Reconstruction: A Systematic Review. Int J Sport Phys Ther. 2022;17(3):334-46.

86. Moesgaard L, Beck MM, Christiansen L, Aagaard P, Lundbye-Jensen J. Effects of Periodization on Strength and Muscle Hypertrophy in Volume-Equated Resistance Training Programs: A Systematic Review and Meta-analysis. Sports Med. 2022.

87. Orange ST, Hritz A, Pearson L, Jeffries O, Jones TW, Steele J. Comparison of the effects of velocity-based vs. traditional resistance training methods on adaptations in strength, power, and sprint speed: A systematic review, meta-analysis, and quality of evidence appraisal. J Sports Sci. 2022:1-15.

88. Vieira JG, Sardeli AV, Dias MR, Filho JE, Campos Y, Sant’Ana L, et al. Effects of Resistance Training to Muscle Failure on Acute Fatigue: A Systematic Review and Meta-Analysis. Sports Med. 2022;52(5):1103-25.

89. Zhang M, Tan Q, Sun J, Ding S, Yang Q, Zhang Z, et al. Comparison of Velocity and Percentage-based Training on Maximal Strength：Meta-Analysis. Int J Sports Med. 2022.
