# Supplementary material for: Preferred Reporting Items for Resistance Exercise Studies (PRIRES): A Checklist Developed Using an Umbrella Review of Systematic Reviews
Source: Sports Med Open. 2023 Dec 1;9:114. doi: 10.1186/s40798-023-00640-1 (PMC10692055; doi:10.1186/s40798-023-00640-1)
Supplement: Supplementary file 7 — Additional file 7. Preferred Reporting Items for Resistance Exercise Studies (PRIRES) Checklist for Protocols. [file 40798_2023_640_MOESM7_ESM.docx]

# Additional File 7: Preferred Reporting Items for Resistance Exercise Studies (PRIRES) Checklist for Protocols

| Topic | Item # | Checklist item | Locations  (NA: not applicable; TBA: to be determined) |
| --- | --- | --- | --- |
| **Exercise Selection, Performance, and Training Parameters (A Flowchart Is Recommended)** | | | |
| Exercise selection | 1a | Provide the name and movement description of every exercise and report the exercises that will be performed in each training session. |  |
|  | 1b | Provide enough information for readers to know whether the exercises will be free-weight or machine-based, and unilateral or bilateral. |  |
|  | 1c | Report the range of motion of each exercise. |  |
| Training frequency and length | 2 | Report the training frequency and length (e.g., 3 sessions/week 🞪 12 weeks). |  |
| Warm-up and movement descriptions | 3 | Report the content of the warm-up and transition (minutes) before the main resistance exercise training. |  |
| Intensity | 4a | Report the absolute training intensity in % 1RM (1 repetition max), #RM, or % #RM (e.g., 75% 1RM, 10RM, or 90% 5RM). |  |
|  | 4b | Report the subjective exercise intensity, e.g., the rate of perceived exertion (RPE). |  |
|  | 4c | If applicable, report the relative training intensity. For example, if a participant performs 5 repetitions of squat with 90 kg and their 5RM is 100 kg, then the relative intensity is 90 kg ÷ 100 kg = 90%. |  |
| Number of repetitions and the set endpoint | 5a | Report the number of repetitions per set. |  |
|  | 5b | Report whether the participants will be asked to train to failure or not. |  |
|  | 5c | Clearly define the set endpoint. For example, the set could be defined to have ended when the participants determine that they cannot complete the next repetition (i.e., self-determined repetition maximum). See Steele et al. (2017)[1] for detail. |  |
| Exercise sequence and the structure of sets | 6a | Exercise sequence:  Report the sequence of all resistance exercises. If the session will include multiple sets, report whether the sequence will be traditional (e.g., squat set 1 → squat set 2 → deadlift set 1 → deadlift set 2) or circuit (e.g., squat set 1 → deadlift set 1 → squat set 2 → deadlift set 2). If other training methods will be used, such as super-set, complex and contrast training, and multiple-joint to single-joint, report the exercise sequence in detail. |  |
|  | 6b | Structure of set:  If an alternative set structure (i.e., cluster set and rest redistribution) will be used, report the method in detail. Describe the intra-set and inter-repetition rest intervals if applicable. |  |
|  | 6c | Number of sets:  Report the number of sets per exercise per training session. |  |
| Rest interval between sets and exercises | 7 | Report the rest interval between sets and exercises. |  |
| Movement tempo | 8a | Report the tempo of resistance exercises in each phase, including but not limited to the durations in eccentric, transition, and concentric phases and the duration between each repetition. If no introduction will be provided, state so. |  |
|  | 8b | If applicable, describe how the tempo will be set. |  |
| Movement velocity | 9 | If applicable, describe how the movement velocity will be recorded. |  |
| Attentional focus | 10 | If applicable, describe the attentional focus strategy, e.g., external focus. |  |
| Concentric or eccentric-focused training | 11a | If non-traditional resistance training (with both concentric and eccentric phases) will be used, report the phase that will be emphasized. |  |
|  | 11b | If applicable, describe the method that will be used to emphasize the concentric/eccentric phase of muscle action, e.g., a longer duration of eccentric phase per repetition. |  |
|  | 11c | If the accentuated eccentric loading method will be used (i.e., the load will be higher during the eccentric phase than during the concentric phase), report the method that will be used to attain the supramaximal eccentric load. |  |
| Drop set | 12 | If applicable, report the number and structure of the drop sets. |  |
| Inter-set intervention | 13 | If an inter-set strategy will be used (instead of having typical rests between sets), report the method. |  |
| **Training Program and Progression** | | | |
| Progression | 14a | Describe in detail the decision rule(s) that will be used for determining exercise progression. |  |
|  | 14b | Describe in detail how the exercise program will progress. |  |
| Autoregulation method | 15 | If autoregulation training will be used, e.g., autoregulatory progressive resistance exercise, rating of perceived exertion, and velocity-based training programs, report the method. |  |
| Programming/ periodization | 16 | Report the resistance training program (i.e., manipulation of parameters such as intensity and volume). If applicable, report the model of programming/periodization, e.g., daily undulating and weekly undulating methods. |  |
| **Exercise Setting** | | | |
| Equipment | 17 | Describe in detail the type of exercise equipment that will be used. |  |
| Location | 18 | Describe the setting in which the exercises will be performed. |  |
| Supervision | 19 | Report whether the resistance training will be supervised or not. If the resistance training will be supervised, report the ratio of instructor to participants. |  |
| Time | 20 | Report the time of training (e.g., 10:00–11:00). |  |
| Blood flow restriction | 21a | If blood flow restriction will be used, describe the pressure modality:  1. Intermittent or continuous (minutes)  2. Pressure (#mm Hg or % of arterial occlusion pressure)  3. Cuff width (cm)  4. Where the pressure was applied  5. The inflator device. |  |
|  | 21b | If blood flow restriction will be used, report how, when, and at what position the arterial occlusion pressure will be determined. |  |
| Elastic resistance training | 22 | If applicable, report the method and equipment that will be used. |  |
| Inertial resistance training | 23 | If applicable, report the method and equipment that will be used. |  |
| Training in hypoxia | 24 | If applicable, report the setting in which the training will occur. |  |
| **Planned vs. Actual Training** | | | |
| Adherence | 25 | Describe how adherence or fidelity to the exercise intervention will be assessed/measured, e.g., via drop-out rate and attendance rate. |  |
| Compliance and deviation | 26 | Describe the deviation from the original exercise protocol that will be reported. |  |

## References

1. Steele J, Fisher J, Giessing J, Gentil P. Clarity in reporting terminology and definitions of set endpoints in resistance training. Muscle & Nerve. 2017;56(3):368-74.
