# Supplementary material for: Preferred Reporting Items for Resistance Exercise Studies (PRIRES): A Checklist Developed Using an Umbrella Review of Systematic Reviews
Source: Sports Med Open. 2023 Dec 1;9:114. doi: 10.1186/s40798-023-00640-1 (PMC10692055; doi:10.1186/s40798-023-00640-1)
Supplement: Supplementary file 2 — Additional file 2. Umbrella Review Reporting Checklist. [file 40798_2023_640_MOESM2_ESM.docx]

# Additional File 2: Umbrella Review Reporting Checklist

| Section/topic | # | Checklist item | Location |
| --- | --- | --- | --- |
| **Title and Abstract** |  |  |  |
| Title | 1 | Identify the reports as an overview of (systematic) reviews, an umbrella review, or a meta-epidemiologic study | Title page |
| Structured summary | 2 | Provide a structured abstract | Title page |
| **Introduction** |  |  |  |
| Rationale | 3 | Specify the rationale for the overview of reviews in the context of an already-formed body of knowledge on the topic | Introduction para. 1-5 & 9; Supplementary File 1 |
| Objectives | 4 | Describe a precise statement of questions | Introduction para. 9 |
| **Methods** |  |  |  |
| Protocol and registration | 5 | Report if an overview protocol was developed and if and where it can be obtained and provide registration information | Section “Registration” |
| Criteria for considering reviews for the overview | 6 | Describe review characteristics and report characteristics for eligibility criteria | Section “Inclusion criteria” and “Exclusion criteria” |
| Information sources | 7 | State all information sources in the search and date least searched | Section “Search strategies and information sources” |
| Search strategy | 8 | Specify full electronic search strategy including any limits used, such as language restriction |  |
| Review selection | 9 | Provide the process for selecting reviews and its relevant details | Section “Data selection and collection process” |
| Additional searches to identify other relevant primary studies | 10 | Report whether and why additional searches were conducted to identify other eligible primary studies | Section “Reporting items” and Figure 1 |
| Data extraction and management | 11 | State the processes of data extraction from included reviews and their relevant details | Section “Data selection and collection process” |
| Data items | 12 | Specify all items overview authors sought (e.g., PICOS, method, results, funding source) | Section “Reporting items” |
| Assessment of methodological quality of included reviews | 13 | Describe methods used for assessing methodological quality and quality of evidence and how this information was used for analyses | NA |
| Data synthesis | 14 | Specify the methods of handling data and their details | Section “Reporting items” |
| **Results** |  |  |  |
| Review selection | 15 | Provide the details of review selection or a flow diagram of the overview process | Results para. 1-2 and Figure 1 |
| Review characteristics | 16 | Describe characteristics of each review (e.g., title, PICOS, number of studies and participants included, assessment of methodological quality of reviews, results of individual reviews) | NA |
| Assessment of methodological quality of included reviews | 17 | Report the results of assessment of methodological quality and quality of evidence of each included review |  |
| Synthesis of results | 18 | Summarize the main findings of the overview. If overview authors undertook data synthesis, present each summary measure with a confidence interval or a credible interval and measures of heterogeneity or inconsistency | Results para. 3 and Table 1 |
| **Discussion** |  |  |  |
| Summary of evidence | 19 | Provide a concise summary of the main findings with the strength and shortcomings of evidence for each main outcome | Discussion para. 1-3 |
| Limitations | 20 | Discuss limitations of the overview of review | Discussion para. 4-6 |
| Conclusion | 21 | Present implications for practice and future research | Conclusions |
| Funding | 22 | Describe source of funding for overview of reviews | Sections “Funding information”, “Sponsor's Role”, and “Competing interests” |

From: Biondi-Zoccai, G. (2016). Umbrella reviews. Evidence synthesis with overviews of reviews and meta-epidemiologic studies (pp. 191-192). Cham, Switzerland: Springer International.
